# Identification of molecular determinants that govern distinct STIM2 activation dynamics

**DOI:** 10.1371/journal.pbio.2006898

**Published:** 2018-11-16

**Authors:** Sisi Zheng, Guolin Ma, Lian He, Tian Zhang, Jia Li, Xiaoman Yuan, Nhung T. Nguyen, Yun Huang, Xiaoyan Zhang, Ping Gao, Robert Nwokonko, Donald L. Gill, Hao Dong, Yubin Zhou, Youjun Wang

**Affiliations:** 1 Beijing Key Laboratory of Gene Resource and Molecular Development, College of Life Sciences, Beijing Normal University, Beijing, P. R. China; 2 Center for Translational Cancer Research, Institute of Biosciences and Technology, College of Medicine, Texas A&M University, Houston, Texas, United States of America; 3 Key Laboratory of Cell Proliferation and Regulation Biology, Ministry of Education, Institute of Cell Biology, College of Life Sciences, Beijing Normal University, Beijing, P. R. China; 4 Department of Cellular and Molecular Physiology, The Pennsylvania State University College of Medicine, Hershey Pennsylvania, United States of America; 5 Kuang Yaming Honors School, Nanjing University, Nanjing, P. R. China; 6 Department of Medical Physiology, College of Medicine, Texas A&M University, Temple, Texas, United States of America; Università di Padova, Italy

## Abstract

The endoplasmic reticulum (ER) Ca^2+^ sensors stromal interaction molecule 1 (STIM1) and STIM2, which connect ER Ca^2+^ depletion with extracellular Ca^2+^ influx, are crucial for the maintenance of Ca^2+^ homeostasis in mammalian cells. Despite the recent progress in unraveling the role of STIM2 in Ca^2+^ signaling, the mechanistic underpinnings of its activation remain underexplored. We use an engineering approach to direct ER-resident STIMs to the plasma membrane (PM) while maintaining their correct membrane topology, as well as Förster resonance energy transfer (FRET) sensors that enabled in cellulo real-time monitoring of STIM activities. This allowed us to determine the calcium affinities of STIM1 and STIM2 both in cellulo and in situ, explaining the current discrepancies in the literature. We also identified the key structural determinants, especially the corresponding G residue in STIM1, which define the distinct activation dynamics of STIM2. The chimeric E470G mutation could switch STIM2 from a slow and weak Orai channel activator into a fast and potent one like STIM1 and vice versa. The systemic dissection of STIM2 activation by protein engineering sets the stage for the elucidation of the regulation and function of STIM2-mediated signaling in mammals.

## Author summary

Calcium ions play a major regulatory role in the physiology and biochemistry of the cell, and thus their levels and activities should be tightly regulated. The stromal interaction molecules (STIMs) are sensors of the calcium levels within the endoplasmic reticulum (ER)—which serves as a major intracellular calcium store—to mediate communication between the ER and the plasma membrane and are regarded as ubiquitous central players of calcium signaling in mammalian cells. STIM2 acts as a slow and weak activator of Orai1 calcium channels on the plasma membrane by direct binding; however, the affinity of STIMs for calcium or how Orai1 channels are activated remain unclear. In this study, we systematically analyzed the molecular determinants that govern the activation of STIM proteins. Adopting protein engineering approaches that enable the relocation of ER-resident STIM proteins at the plasma membrane, we determined the calcium affinities of STIMs under physiological conditions in mammalian cells. We identified a critical position within STIMs, which defines their distinct resting states and activation kinetics, as well as the efficacy to activate Orai1 channels. These findings shed new light on how STIM2 can efficiently respond to small changes within the ER lumen to regulate calcium homeostasis and signaling in mammalian cells.

## Introduction

Store-operated Ca^2+^ entry (SOCE) is a major Ca^2+^ influx pathway that is crucial for many types of cellular functions [1–4]. SOCE is mediated by stromal interaction molecule 1 (STIM1) and STIM2, dynamic Ca^2+^ transducers localized at junctions between the endoplasmic reticulum (ER) and plasma membrane (PM) [5]. The ER luminal domains of STIMs contain a Ca^2+^-binding EF-hand motif, a hidden non-Ca^2+^–binding EF hand (EF), and the sterile alpha motif (SAM) domain (EF-SAM) [6, 7], together serving as ER Ca^2+^ sensors. The cytosolic regions of STIMs comprise the STIM-Orai–activating region (SOAR) [8] or Ca^2+^-release–activated Ca^2+^ (CRAC)-activating domain (CAD) [9] and function as activators of Orai Ca^2+^ channels situated in the PM [1, 10]. STIM2 and its splice variants play important roles in fine-tuning SOCE by diversifying functional Orai–STIM combinations [5, 11–13] in the immune and nervous systems (reviewed in [14, 15]). Abnormalities in STIM2-mediated SOCE have been linked to diminished salivary fluid secretion, impaired sweat secretion, Alzheimer’s disease, and Huntington's disease in mouse models [13, 16–21]. Although considerable progress has been made in understanding STIM1-mediated SOCE [1, 5, 10, 22], the role of the STIM2 molecule has been largely ignored and is consequently less well known.

Two crucial issues regarding STIM2 activation remain to be addressed immediately. First, the Ca^2+^ binding affinity of the EF-SAM of STIM2 should be determined in cellulo. The applicability of in vitro determinations and in situ estimations to addressing this issue is limited [6, 23]. In vitro measurements are carried out in a non-membrane–like environment using purified recombinant proteins under nonphysiological conditions and hence may not reflect the true behavior of STIMs embedded in the ER membrane [24]. The in situ estimations are based on calculated values of resting Ca^2+^ levels within the ER lumen, a subcellular compartment that is difficult to access and with the measurements subject to large variations depending on which Ca^2+^ indicators are used [25–29]. As a result, previous in vitro determinations and in situ estimations showed discrepancies in the cooperativity of Ca^2+^ binding [6, 23, 30]. In vitro measurements showed that the Ca^2+^ binding behavior of STIM1 is temperature dependent [31] and that the Ca^2+^ affinities measured under room temperature showed no difference between STIM1 and STIM2 [24], which is inconsistent with results from in situ measurements [23, 26, 30]. Thus, a better approach is needed to determine the Ca^2+^ affinities of STIMs, ideally enabling in cellulo measurements to reconcile the discrepancies in the reported values.

Second, it is unclear how STIM2, a slow Orai activator [32–34], gets activated and mediates Ca^2+^ influx to compensate for small fluctuations in the Ca^2+^ levels within the ER lumen [5, 23]. The formation of puncta and the development of I_CRAC_ current or SOCE through STIM1-activated Orai1 channels have been used as indicators of the activation status of STIM1 [5]. However, these approaches are not appropriate for the dissection of STIM2 activation. As a weak Orai1 activator [35–37], STIM2 induces only small Ca^2+^ influx through Orai1, and the I_CRAC_ or SOCE measurements are not sufficiently sensitive to describe its activation status. Further, STIM2 is always partially active, forming constitutive puncta and constantly inducing Ca^2+^ flux through Orai1 channels at rest [23, 32, 33]. This makes it difficult to use puncta or current measurements to gauge its degree of activation following store depletion. A new tool is thus clearly required to uncover the mechanistic details of STIM2 function.

To tackle these challenges, we designed a set of molecular tools to report the activation status of STIM2 in situ in real time based on a two-component FRET biosensor recently developed by us [38]. We bypassed the accessibility issue of the ER luminal Ca^2+^-binding EF-SAM region by redirecting engineered STIM constructs to the PM so that the luminal domain faced toward the extracellular space. This allowed us, for the first time, to determine the apparent Ca^2+^ affinities of STIM constructs in cellulo by simply changing the Ca^2+^ levels in the extracellular medium. We also engineered a series of ER-resident STIM1/STIM2 chimeric constructs that stayed quiescent at rest, which enabled an accurate dissection of the contribution of the individual key structural elements of STIM2 to the protein’s activation kinetics and dynamics. With these FRET-based probes, we identified E470 in SOAR2 as a critical residue that rendered SOAR/CAD more activated at rest and accounted for a narrower dynamic range for STIM2 activation compared with STIM1. These novel findings well explained a long-standing puzzle in the field: how STIM2 is able to efficiently respond to minor changes in ER luminal Ca^2+^ levels. Overall, our systematic analysis provided new insights into STIM2 activation dynamics and kinetics.

## Results and discussion

### A novel protein-engineering strategy to expose the luminal domain of STIM toward the extracellular space

**Fig 1:**
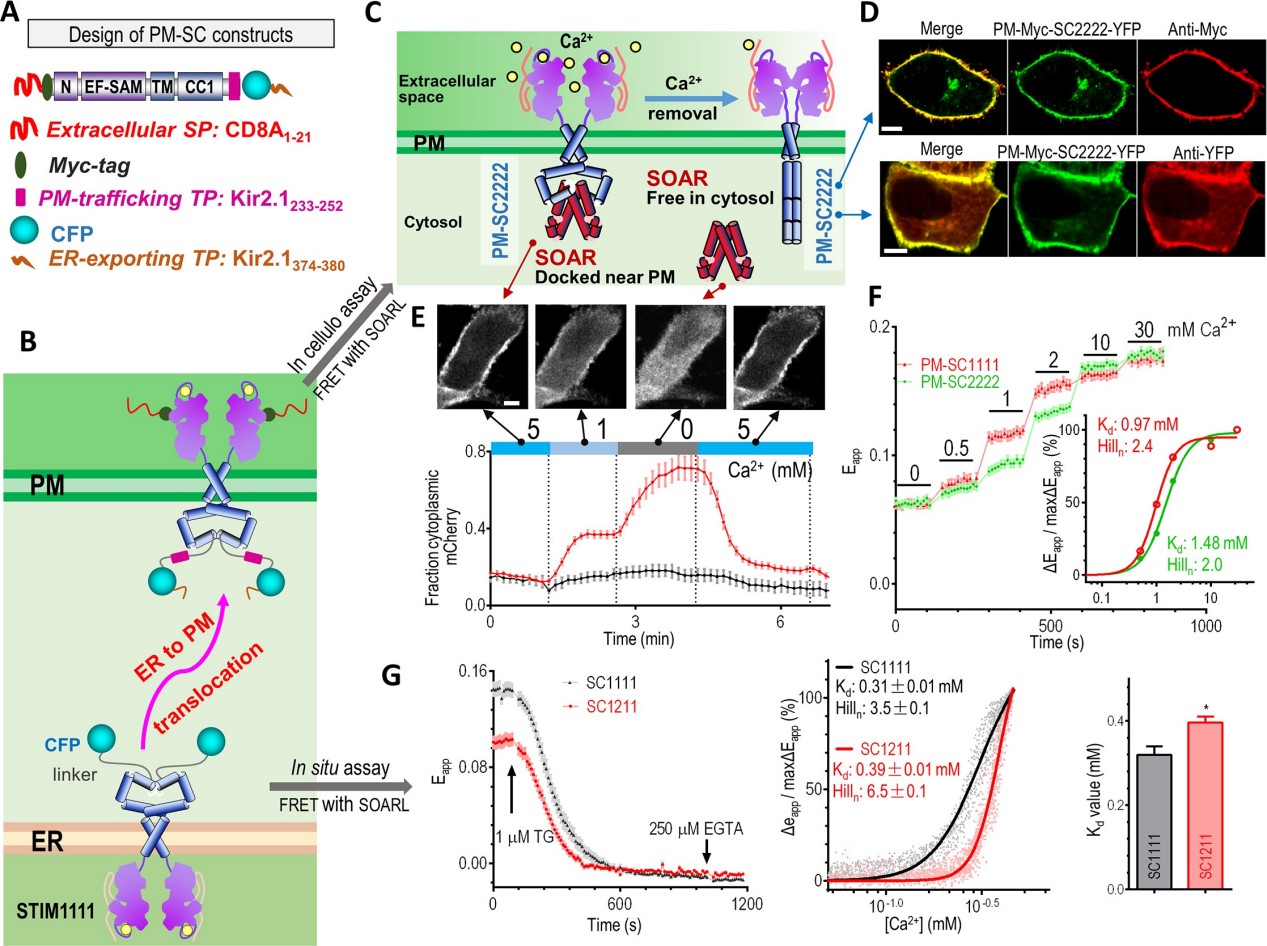
In cellulo and in situ determination of the apparent Ca^2+^ affinities of STIMs using engineered nanosensors. (A) Diagram showing the design of the engineered PM-anchoring SCs. Myc tag and three SPs or TPs that aided ER extrusion and PM export of the SCs were engineered into SCs. (B) A cartoon of the different cellular distributions of SCs (ER) and PM-anchoring SCs (PM). (C) A diagram of the design of the PM-localized nanosensors for the quantification of Ca^2+^ affinities of STIM in cellulo. A sensor has two components: a YFP-tagged cytosolic SOAR/CAD or SOAR1L (STIM1_343-491_) domain and a CFP-tagged, PM-anchoring SC localized in the PM, with the Ca^2+^-sensing EF-SAM facing the extracellular space. Sensing of the changes in extracellular Ca^2+^ levels by the EF-SAM of the PM-anchoring SC initiates conformational changes in the SC to disrupt its interaction with SOAR1L. This results in the redistribution of SOAR within cells and impacts the FRET signals between the nanosensor pair. More details of the design strategy are given in S2 Fig. (D) Typical confocal images of cellular distribution of PM-SC2222-YFP expressed in HeLa cells (representative for at least 38 cells). The localization of SC2222 is revealed both by antibodies in live cells immunostaining without PM permeabilization against its N-terminal Myc tag (top right image, middle images) and coexpressed YFP-nanobody mCh-tagged LAG9 distribution around the PM (bottom right image). Scale bar, 10 μm. (E) Upon co-transfection with PM-localized SC1111-CFP (PM-SC1111) in HeLa cells, the cellular distribution of mCh-CAD changes with changing extracellular Ca^2+^ concentrations. Top images: typical cellular distribution of mCh-CAD in bath solutions with different Ca^2+^ content (approximately 55 cells examined). The Ca^2+^ concentration is stated underneath each image. Bottom trace: Changes in the relative ratio of cytoplasmic mCh fluorescence to PM mCh fluorescence with changes in extracellular Ca^2+^ concentrations. The black trace shows the CAD signal when not coexpressed with PM-SC1111; the red trace represents the CAD signal when coexpressed with PM-SC1111. (F) FRET responses between YFP-SOAR1L and engineered PM-STIM_CC1_–CFP constructs. Left: representative traces; right: dose response curves (*n* = 3, more than 43 cells examined in each group). (G) In situ determination of Ca^2+^ affinities of STIM constructs. In HeLa SK cells coexpressing R-CEPIA1er, YFP-SOAR1L, and SC1111-CFP or SC1211-CFP, ER Ca^2+^ levels and FRET signals between SCs and SOAR1L were monitored simultaneously. Left: Typical traces of the rest state and TG-induced responses for FRET signals between YFP-SOAR1L and SC1111-CFP or SC1211-CFP. Middle: Typical relationships between the ER Ca^2+^ levels and the relative changes in E_app_ signals, calculated from left trace. Solid lines are fits of data points using the Hill equation. Right: Statistical analysis for the Ca^2+^ affinities of the ER-distributed STIM constructs (*n* = 3, *P < 0.05, paired *t* test). Individual numerical values underlying (E), (F), and (G) may be found in S1 Data. CAD, CRAC-activating domain; CC1, coiled-coil 1; CRAC, Ca^2+^-release–activated Ca^2+^ current; CFP, cyan fluorescent protein; EF-SAM, EF-hand and sterile alpha motif domain; ER, endoplasmic reticulum; FRET, Förster resonance energy transfer; mCh, mCherry; PM, plasma membrane; SAM, sterile alpha motif; SC, STIM_1-CC1_ construct; SOAR, STIM-Orai–activating region; SK, STIM1 and STIM2 double knockout; SP, signal peptide; STIM, stromal interaction molecule; TG, thapsigargin; TP, target peptide; YFP, yellow fluorescent protein.

To circumvent the difficulties of accessing the ER lumen and determining the ER Ca^2+^ levels, we first engineered STIM1 and STIM2 proteins to relocate them to the PM, with their luminal region facing the extracellular space. We replaced the original signal peptide (SP) of STIM with the SP derived from CD8A_1-21_ and introduced a PM-trafficking target peptide (TP) (Kir2.1_233−252_) and an ER-exporting TP (Kir2.1_374−380_) at the C terminus of STIM (Figs 1A–1C and S1 and S2A). Through several rounds of optimization (S2B Fig), the resulting PM-Myc-STIM2_1-CC1_ or PM-STIM_1-CC1_ construct tagged with yellow fluorescent protein (YFP) (see S1E and S1F Fig for nomenclature details of the engineered constructs) showed PM-like distribution in HeLa cells (Fig 1D, middle). The coiled-coil 1 (CC1) of PM-SC2222-YFP faced the cytosol because the YFP tag could only be recognized by an engineered nanobody that binds to green fluorescent protein (GFP) or YFP (i.e., mCherry [mCh]-tagged LAG9) in live cells (Fig 1D, bottom row). By contrast, the N terminus of the engineered STIM faced toward the extracellular space because the N-terminal Myc tag was detected by immunostaining of live cells without PM permeabilization (Fig 1D, top row; S2A and S2B Fig). Moreover, the cytosolic mCh-CAD migrated toward PM in HeLa cells co-transfected with PM-SC2222-YFP in the presence of 2 mM Ca^2+^ (S2C Fig), while lowering the extracellular Ca^2+^ concentration caused cytosolic dispersion of mCh-CAD. This strongly implied that PM-SC2222 retained its native structure even after translocation to the PM. Hence, this approach constitutes a convenient engineering strategy of forcing the trafficking of transmembrane proteins originally embedded in the ER membrane to the PM. After relocation to the PM, the proteins retained their proper membrane topology and exposed the otherwise inaccessible luminal domains to the extracellular space. Effectively, this overcame the major impediment to the biophysical and electrophysiological studies of ER-resident ion channels and transducers. Specifically, the engineered ER-to-PM trafficking constructs allowed, for the first time, a precise and facile manipulation of the extracellular Ca^2+^ levels in the vicinity of the extracellular EF-SAM.

We then coexpressed mCh-CAD and PM-SC1111-CFP in HeLa cells and examined whether the association of CAD and CC1 of PM-SC1111 depended on the extracellular Ca^2+^ levels (Fig 1E). With the extracellular Ca^2+^ levels in a millimolar range, the cytosolic mCh-CAD displayed a PM-like decoration (Fig 1E, leftmost image), indicating its specific interaction with PM-SC1111 (Fig 1C, left). The latter adopts a resting inactive conformation in its Ca^2+^-bound form [5]. After switching to a nominally Ca^2+^-free extracellular solution, PM-docked CAD molecules rapidly dissipated into the cytosol (Fig 1E, second image from the right), indicating dissociation from PM-SC1111. These observations clearly established that the EF-SAM of PM-SC1111 sensed the fluctuation of extracellular Ca^2+^ levels, similarly to sensing Ca^2+^ depletion within the ER lumen (“store depletion”), and could faithfully phenocopy the Ca^2+^-dependent switch between active and inactive conformations (Fig 1C). This process was fully reversible because the cytosolic CAD immediately redecorated the PM when extracellular Ca^2+^ was replenished to the mM range (Fig 1E, right panel).

### Both in cellulo and in situ measurements revealed that STIM2 constructs have lower apparent Ca^2+^ affinities

Following this, we used a FRET assay to precisely determine the Ca^2+^-binding affinities of STIM in cellulo. In the assay, YFP-SOAR was the acceptor and PM-SC1111-CFP was the donor, which allowed the characterization of the CC1–SOAR interactions in response to alterations in extracellular Ca^2+^ levels. We used a slightly modified SOAR1 variant, SOAR1L (STIM1_343-491_), instead of SOAR1 (STIM1_344-442_) (S1G Fig) because of its superior performance in FRET experiments with PM-anchoring SCs. To avoid artifacts induced by endogenous STIM1 or STIM2 molecules and the filling status of the ER Ca^2+^ stores, the FRET experiments were performed in STIM1 and STIM2 double knockout (SK) HeLa cells (Fig 1F). The obtained apparent K_d_ value of STIM2 was lower than that of STIM1. Interestingly, both values were in the mM range (STIM1: 0.97 ± 0.02 mM and STIM2: 1.48 ± 0.02 mM; Fig 1F), much higher than previous reports (1.0 versus 0.2 mM for STIM1; 1.5 versus 0.4 mM for STIM2) [23, 24]. Furthermore, these results would predict both STIM1 and STIM2 being constitutively active at rest. Such a prediction contradicts current knowledge about STIM [5, 33] and is not consistent with our own observations showing that only STIM constructs with STIM2-EF-SAM were constitutively active (S3A Fig). Since other engineered PM-anchoring SCs (S3B Fig) showed a similar trend, we thus checked whether these “abnormal” values were artifacts caused by protein engineering. A minor portion of cells (approximately 20%–30%) expressing unengineered SCs with the STIM2-CC1 domain showed some PM-like distribution, and results from these cells showed that even unengineered SCs with PM-like distribution also bear similarly high K_d_ values (S1C and S1D and S3C Figs). We also performed in cellulo measurements with bath solutions that contained high K^+^ (140 mM) and low Na^+^ (10 mM) to mimic the ER-like ionic and electric environment and obtained values no different from those done with regular extracellular solution. These results indicate that the high K_d_ values we obtained were not artifacts caused by low K^+^ concentration or negative membrane potentials induced by regular extracellular solutions. Thus, our in cellulo results showed that STIM constructs with their EF-SAM facing extracellular space do have much lower affinities for Ca^2+^ than previous in vitro and in situ measurements [23, 33].

This prompted us to perform in situ calibrations using the ER-localized SC constructs and R-CEPIA1er (an ER Ca^2+^ indicator that is more sensitive than D1ER [25]) [26], as previously described (Figs 1G and S3D and S3E) [23, 26]. Using the STIM1_CC1_-SOAR1L FRET as a readout for STIM activation [38], the in situ approach done in HeLa SK cells showed that SC constructs with STIM1-EF-SAM or STIM2-EF-SAM both bind Ca^2+^ with a high cooperativity (Hill_n_: 3.5 ± 0.1 for SC1111, 6.5 ± 0.1 for SC1211), similar to previous in situ results (Hill_n_ around 4) [23, 25, 30]. The results also showed that the Ca^2+^ dissociation constants (K_d_) of STIM1_EF-SAM_ and STIM2_EF-SAM_ were 0.31 ± 0.04 mM and 0.42 ± 0.06 mM, respectively. Using STIM1 puncta as readouts, we also found a similar Ca^2+^ affinity for full-length STIM1 (0.33 ± 0. 2 mM). All these in situ values were similar to the previously reported ones. Thus, our in situ results again validated the robustness of the STIM_CC1_ and SOAR1L FRET signals as readouts for STIM activation. Overall, obtained with the same FRET-based readout (Fig 1B), results from our in cellulo and in situ measurements both agree that the Ca^2+^ affinity of STIM2 is lower than that of STIM1 (Fig 1F and 1G).

Our in cellulo data and in situ results have important implications. The discrepancy between our in cellulo results and previous in vitro data indicates that the EF-SAM, associated with the membrane under physiological conditions, may behave differently than isolated recombinant proteins in an aqueous solution in vitro [24]. Indeed, STIM EF-SAM fragments bind Ca^2+^ with a stoichiometry of 1 in vitro [13, 19], indicating that isolated EF-SAM has no cooperativity in Ca^2+^ binding. While the in cellulo data showed Hill numbers around 2 (Hill_n_: 2.7 ± 0.3 for STIM1; 2.3 ± 0.03 for STIM2; *n* = 4), indicating that membrane-associated STIM-EF-SAM has some cooperativity. The in cellulo Hill numbers were consistent with the notion that STIMs function as dimers (reviewed in [35]). When measured in situ, the Hill numbers significantly increased to around 4 (Fig 1F versus Fig 1G) (*P* < 0.001, *n* = 4, Student *t* test) [23, 25, 30], indicating that STIM proteins on ER membrane have a much higher cooperativity in Ca^2+^ binding. Since the in cellulo data and in situ results were obtained with the same FRET readout in the same type of cells, the observed differences in Ca^2+^ bindings thus clearly indicated the existence of possible additional modulators or post-translational modifications of STIM within the ER lumen, providing an explanation for current discrepancies between in situ and in vitro results in the literature. Recently, it was shown that STIM1 glycosylation at residues N131 and N171 substantially reduced its Ca^2+^ affinity [39]. However, no other protein regulators affecting the Ca^2+^ sensitivity of STIMs within the ER lumen have been reported to date. Further follow-up studies are needed to identify factors that alter the Ca^2+^-binding behavior of STIM proteins.

Collectively, the differences between our in cellulo and in situ results indicate the existence of possible modulators for STIM within the ER lumen, explaining why previous in situ results differ from in vitro ones. In the meantime, our in cellulo and in situ results both reveal differences in the Ca^2+^ binding affinities of STIM1 and STIM2.

### STIM2-TM slows down the kinetics of STIM2 activation

After determining the Ca^2+^ affinity of the luminal STIM2-EF-SAM that defined its partial activation status at rest, we then addressed the kinetics of its activation. We first examined the applicability of FRET-based nanoprobes [38] for monitoring the STIM activation kinetics in situ. When the N terminus of SC1111 was replaced with that of STIM2 (SC2111), the rate of ionomycin-induced decreases in apparent FRET efficiency (E_app_) signals between SC1111 and SOAR1 was significantly reduced (SC1111: –0.0020 ± 0.0001 ΔE_app_/s versus SC2111: –0.0009 ± 0.0001 ΔE_app_/s, *n* = 3, *t* test, *P* < 0.0001). This was consistent with previous findings from whole-cell patch clamping [32] and confirmed that the FRET-based nanoprobe could indeed be used to determine the STIM activation kinetics.

**Fig 2:**
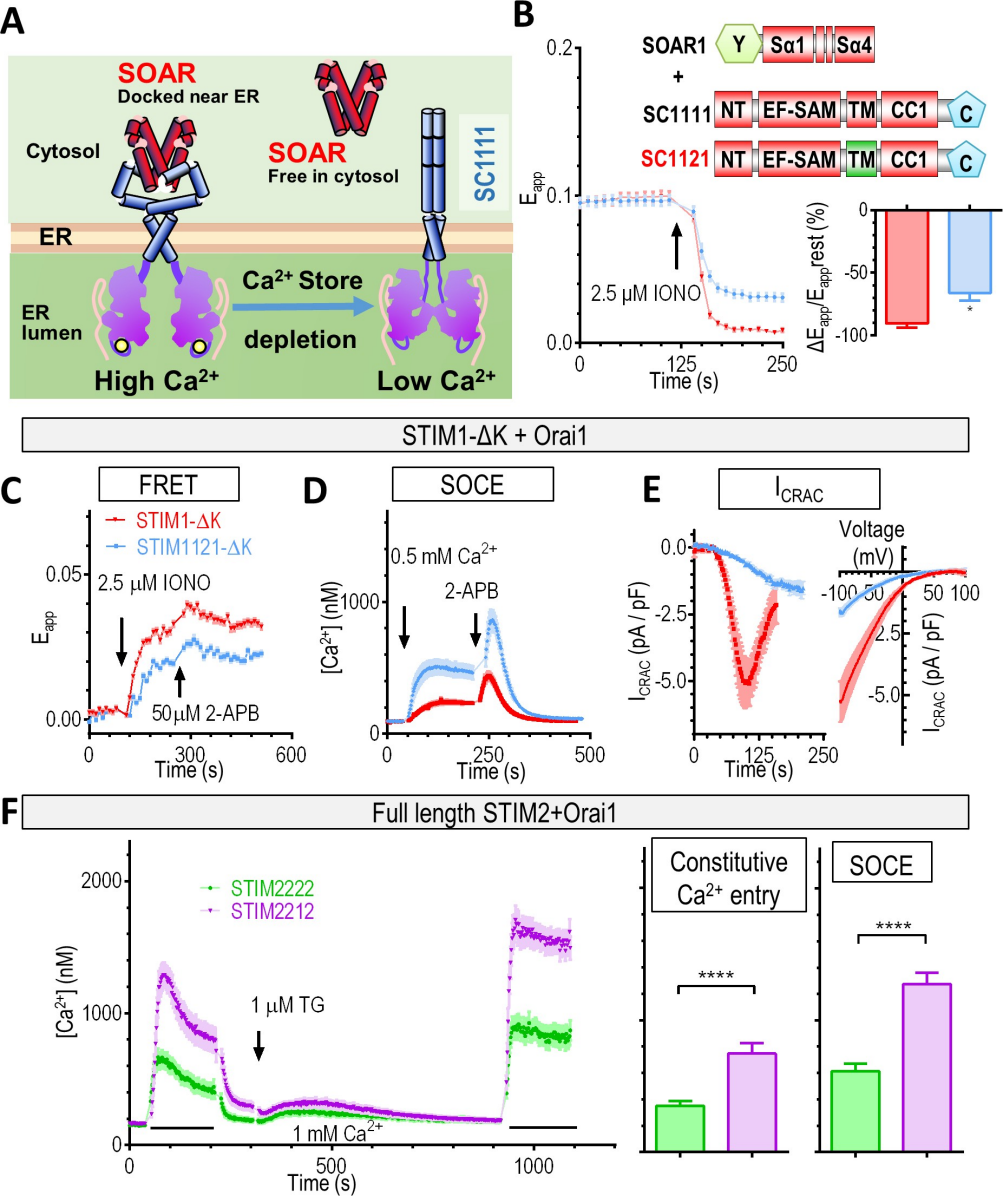
The STIM2-TM domain activates the cytosolic region of STIMs less efficiently than that of STIM1. **(**A) A diagram of colocalization of the ER-localized SC1111 and SOAR under resting or Ca^2+^ store-depleted condition. (B) When the STIM1-TM is replaced with that of STIM2, IONO-mediated Ca^2+^ store depletion induced slower and smaller decrease in the FRET signals between SC1211 and SOAR than SC1111 and SOAR. Left: typical traces; right: statistics of the rate of FRET decrease (*n* = 4, *P < 0.02, *t* test; –90.47% ± 6.66% versus –66.13% ± 11.93%). (C–E) HEK293-Orai1-CFP stable cells transiently expressing STIM1111-ΔK (STIM1-ΔK)-YFP or STIM1121-ΔK-YFP. (C) Typical FRET signals of STIM1121-ΔK and Orai1 before and after Ca^2+^ store depletion. IONO induced a smaller FRET increase in STIM1121-ΔK–expressing cells than in STIM1-ΔK–expressing cells (0.028 ± 0.001 versus 0.018 ± 0.001, *n* = 4, ****P < 0.0001, *t* test). (D) Typical SOCE responses as indicated by Fura-2 imaging. TG-induced SOCE responses were reduced in cells expressing STIM1121-ΔK (358.7 ± 49.35 versus 117.7 ± 12.94, *n* = 4, ****P < 0.0001, *t* test). Cells were pretreated with 1 μM TG for 10 min to deplete the ER Ca^2+^ stores before the measurements. TG was also present during the recordings. (E) Average whole-cell I_CRAC_ recordings. Left: average time course of whole-cell I_CRAC_ currents measured at –100 mV. Currents mediated by STIM1121-ΔK developed significantly more slowly than control (time to peak, 72 ± 7 s versus 147 ± 9 s, *n* = 7, ****P < 0.0001, *t* test), with the maximal current density also reduced (–5.5 ± 0.8 pA/pF versus –1.5 ± 0.1 pA/pF, *n* = 7, *****P < 0.0004, *t* test). To aid comparison, the individual traces were aligned according to the onset of I_CRAC_ (+36 s). Right: average I–V relationships at the peak of I_CRAC_. (F) Ca^2+^ responses of HEK293-Orai1-CFP stable cells transiently expressing STIM2222-ΔK (STIM2-ΔK)-YFP or STIM2212-ΔK-YFP. Left: representative traces. Middle: statistics for constitutive Ca^2+^ entry, Right: statistics for SOCE (*n* = 3, ****P < 0.0001, *t* test). Individual numerical values underlying (B–F) may be found in S1 Data. 2-APB, 2-Aminoethoxydiphenyl borate; CC1, coiled-coil 1; CRAC, Ca^2+^-release–activated Ca^2+^ current; ER, endoplasmic reticulum; FRET, Förster resonance energy transfer; HEK293, human embryonic kidney 293 cells; IONO, ionomycin; I–V, current–voltage; NT, N terminus; SAM, sterile alpha motif; SC, STIM_1-CC1_ construct; SOAR, STIM-Orai–activating region; SOCE, store-operated Ca^2+^ entry; STIM, stromal interaction molecule; TG, thapsigargin; TM, transmembrane region; YFP, yellow fluorescent protein.

We subsequently examined the effect of swapping the STIM2 transmembrane region (STIM2-TM) for SC1111 on its activation kinetics (Fig 2A and 2B). Replacing STIM1-TM with STIM2-TM domain (SC1121) did not affect the basal E_app_ between SC constructs and SOAR1, while the ionomycin-induced rate of changes in E_app_ (ΔE_app_) (–0.0033 ± 0.0011 versus –0.0022 ± 0.0009 ΔE_app_/s) and extent of E_app_ decrease was significantly reduced (–90.47% ± 6.66% versus –66.13% ± 11.93%) (Fig 2B). This indicated that following Ca^2+^ store depletion, STIM2-TM transduced the depletion signal across the ER membrane to the cytosolic portion of STIMs more slowly and less efficiently, resulting in slower and reduced release of SOAR molecules from the STIM CC1.

We then confirmed the findings in the context of full-length STIMs with or without the PM anchoring lysine-rich (K) domain [8, 9, 40]. PM tethering by the K region was recently shown to accelerate the activation of STIM1 molecules [41]. To avoid possible complications caused by PM tethering, we thus first examined the effects of TM domain swapping with STIM-ΔK constructs. The coupling of STIM1 harboring STIM2-TM (STIM1121-ΔK) with Orai1 was reduced (Fig 2C) and, functionally, its ability to induce SOCE was significantly impaired (Fig 2D). We then directly measured the activation kinetics with whole-cell current recordings. I_CRAC_ mediated by STIM1121-ΔK indeed developed significantly more slowly than that mediated by STIM1-ΔK (Fig 2E, traces in the left panel). Consistent with FRET and SOCE measurements (Fig 2B and 2D), the magnitude of the peak current density was also significantly reduced in STIM1121-ΔK–expressing cells (Fig 2E, traces in the right panel). Thus, exchanging STIM2-TM for STIM1-ΔK rendered the activation of STIM1-ΔK slow and inefficient. Conversely, STIM2 harboring STIM1-TM (STIM2212) activated Orai1 and induced Ca^2+^ influx more efficiently both at rest and after store depletion than STIM2 (Fig 2F). Among the three TM residues that are different between STIM (STIM1: M215, V217, I231 versus STIM2: I306, T308, T324), we do not know which of them are more important for defining their activation kinetics. Since the two hydrophobic residues (STIM1 V217 and I231) that are involved in the packing of STIM1-TM [42] are changed to two polar threonines in STIM2, we speculate that these two polar residues might compromise the packing of STIM2-TM and subsequent activation events. Further research is needed to clarify this.

Nevertheless, these observations suggested that STIM-TM plays a role in defining the kinetics of STIM activation and that STIM2-TM is a weak transducer of the ER Ca^2+^ signals. Collectively, we conclude that the luminal domains or EF-SAMs of STIM define its Ca^2+^ sensitivity, which determines whether there will be constitutive Ca^2+^ influx at rest (S4A and S4B Fig versus S4C and S4D and S3A Figs), and that the entire luminal and TM domain of STIM controls the kinetics of its activation upon store depletion.

### SOAR2 domain determines a distinct activated configuration of STIM2 at rest

Once the conformational changes of STIM1 trigged by Ca^2+^ store depletion propagate across the ER membrane, the cytosolic region of STIM1 adopts a more activated configuration by overcoming intramolecular clamping mediated by the CC1–SOAR interaction [5, 38, 43–45], moving the SOAR region of STIM1 from the vicinity of the ER membrane [38, 46, 47] across the ER–PM junctions to activate Orai1 channels on the PM to evoke Ca^2+^ influx [5]. However, it remains unclear whether the cytosolic region of STIM2 undergoes similar dynamic changes after store depletion. We addressed this question by generating a series of chimeric STIM nanoprobes to map the critical protein regions involved in the intramolecular conformational switch (S1 Fig).

**Fig 3:**
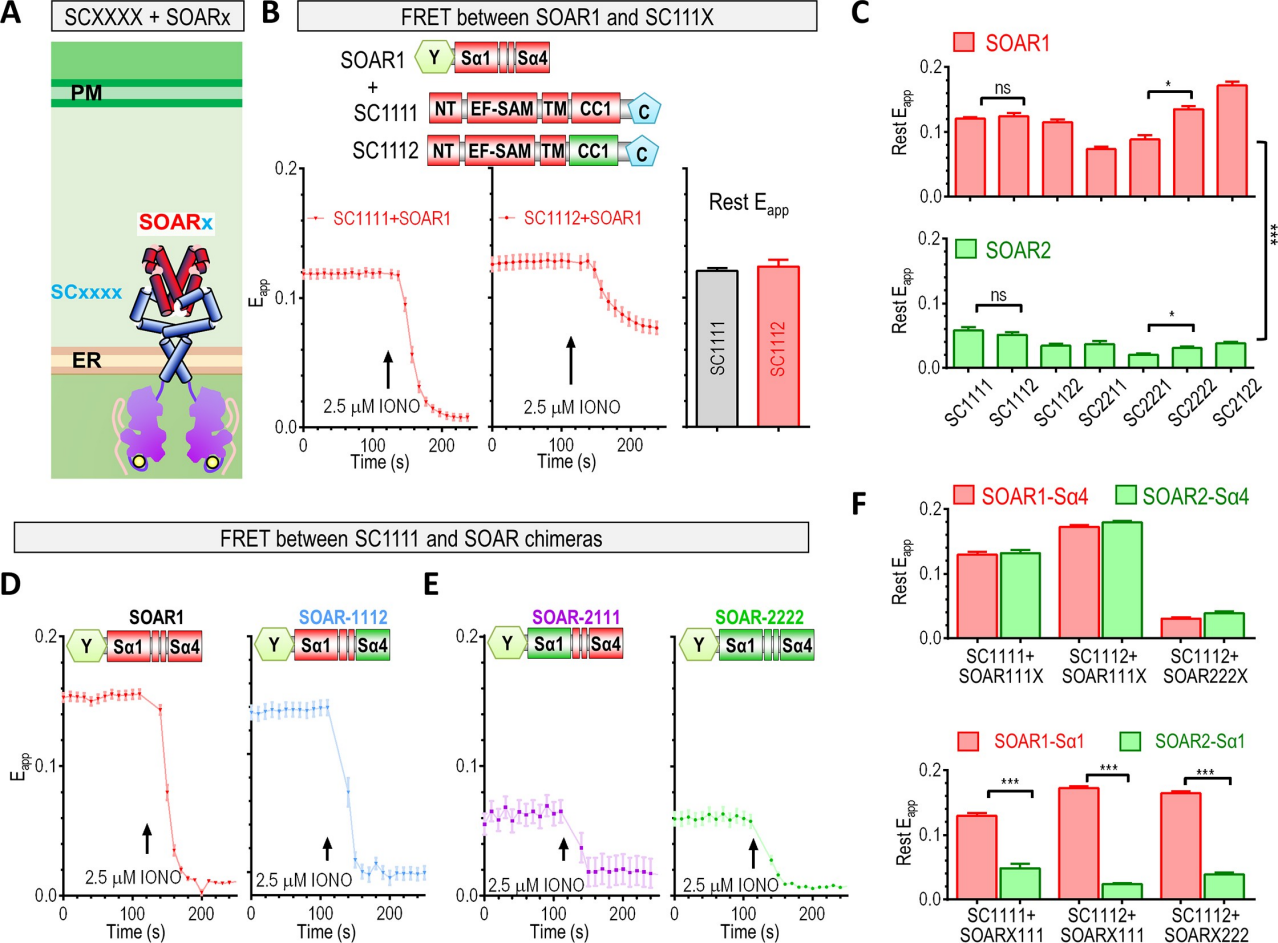
SOAR α1 helix defines its ability to interact with the STIM-CC1 region. (A–C) Comparative analysis of the effect of CC1 or SOAR α1 exchange on the resting FRET signals between STIM_1-CC1_ and SOAR molecules. (A) A diagram showing the FRET-based nanosensors used. (B) The effect of CC1 exchange on FRET signals between SC111x and SOAR molecules. Left and middle: typical traces; right: statistical analysis for the resting FRET reading (*n* = 3, *P* = 0.50, *t* test). (C) Statistical analysis showing the effects of CC1 or SOAR exchange on the resting FRET signals (E_app_) between STIM_1-CC1_ and SOAR molecules (*n* = 3, *P < 0.05, ***P < 0.001, *t* test). (D–F) Comparison of the effects of SOAR α1 or α4 exchanges on FRET signals between STIM_1-CC1_ and SOAR molecules: (D) exchange of the SOAR α4 region; (E) exchange of the SOAR α1 region. (F) Statistics showing the effects of SOAR α1 or α4 exchanges on resting FRET signals (E_app_) between SOAR and SCs. Top panel: the effect of SOAR α4 exchange; bottom panel: the effect of SOAR α1 exchange (*n* = 3, ***P < 0.001, *t* test). Individual numerical values underlying (B)–(F) may be found in S1 Data. C, cyan fluorescent protein; CC1, coiled-coil 1; ER, endoplasmic reticulum; FRET, Förster resonance energy transfer; IONO, ionomycin; ns, not significant; NT, N terminus; PM, plasma membrane; SAM, sterile alpha motif; SC, STIM_1-CC1_ construct; SOAR, STIM-Orai–activating region; STIM, stromal interaction molecule; TM, transmembrane region; Y, yellow fluorescent protein.

We first asked whether the cytosolic region of STIM2 was autoinhibited by an intramolecular clamp (Fig 3A). Consistent with the existence of constitutive puncta formed by chimeric full-length STIM1122 (S4A Fig versus S4B Fig), the basal FRET signals between sensors containing STIM2-CC1 and SOAR2 were considerably lower than those of STIM1 (top traces, S4E Fig versus S4F Fig [*n* = 3, ****P < 0.0001, *t* test]; S4G Fig versus S4H Fig [*n* = 3, ****P < 0.0001, *t* test]), indicating that the STIM2-CC1 failed to retain SOAR2 near the ER membrane as STIM1 did. This suggested that CC1 of STIM2 partially lost the capability to interact with SOAR2. We validated this observation by generating an STIM1 chimera in which the cytosolic region was swapped for that of STIM2 (STIM1122). At rest, even though cells expressing STIM1122 had no constitutive Ca^2+^ influx, the STIM1122 construct showed discernible punctate distribution in the absence of store depletion (S4B Fig), indicating that the cytosolic region of STIM2 adopts a distinct partial activated configuration. This is consistent with a recent report showing that the cytosolic fragments of STIM2 are less well folded [48]. It is well established that the location of the SOAR1 region in STIM1 is critically defined by the folding status of the STIM1 cytosolic region. At rest, the cytosolic region of STIM1 is well folded, keeping SOAR1 close to the ER. Once activated, the cytosolic region becomes extended, bringing SOAR1 close to the PM [49]. We reasoned if the location of SOAR2 in STIM2 were determined by the same principles as those for SOAR1 in STIM1, then the less-well–folded cytosolic region of STIM2 would indicate that its SOAR2 domain was located farther away from ER and closer to the PM.

We then proceeded to identify the structural elements responsible for the weak CC1–SOAR interaction within STIM2. To do that, we analyzed the effect of subdomain swapping on the resting FRET signals of chimeric nanosensors (Fig 3A–3C). The result indicated that the CC1 domain of STIM2 restricted and slowed down the release of SOAR1 from CC1 after store depletion (–0.0032 ± 0.0005 versus –0.0012 ± 0.0006 ΔE_app_/s) (Fig 3B). Further experiments on more chimeric constructs failed to detect this trend, thus indicating the CC1 domain has a minor role in determining the kinetics of STIM activation. Similarly, swapping of the CC1 domain had minimal effect on the resting FRET signals between SC constructs and SOAR molecules (Fig 3C). These observations ruled out the possibility that STIM-CC1 is a major determinant of STIM activation. In contrast, swapping SOAR1 for SOAR2 significantly and consistently reduced the resting FRET signals (Fig 3C), thereby unequivocally establishing that the SOAR region shaped the initial configuration of the cytosolic region of STIM2 molecules at rest. The lower basal FRET signals between STIM-CC1 and SOAR2 indicate that the SOAR2 region is farther away from CC1 in full-length STIM2 (Fig 3C). And since the STIM-CC1 domain directly anchors on the ER membrane via the STIM-TM domain, these results thus indicate that the SOAR2 region is farther away from the ER at rest. As domains localized between the ER and PM membranes, SOAR2 being farther away from the ER membrane would indicate that it is closer to the PM.

Hence, unlike STIM1, the SOAR2 domain in STIM2 docks near the ER membrane less well, enabling for STIM2 a distinct partial activated configuration at rest.

### E470 in the α1 helix of SOAR2 is a major determinant of its weak interaction with STIM2-CC1

We mapped the domains within SOAR2 responsible for its weak interaction with CC1 by examining the effect of swapping the SOAR subdomains on the resting FRET signals of the chimeric nanosensors [37, 38]. The results showed that swapping the α4 helix of SOAR had no effect on basal FRET signals between SC constructs and SOAR molecules (Fig 3D). On the contrary, swapping the α1 helix from SOAR2 into SOAR1 resulted in greatly diminished basal FRET with SC1111, similar to that of SOAR2 (Fig 3E) and much lower than that of SOAR1 (Fig 3D). Further results also confirmed that only the swapping of α1 helix would change basal FRET (Fig 3F). Together, these results revealed that the α1 helix of SOAR is the region determining the basal FRET between chimeric SOAR and SC constructs (Fig 3D–3F).

**Fig 4:**
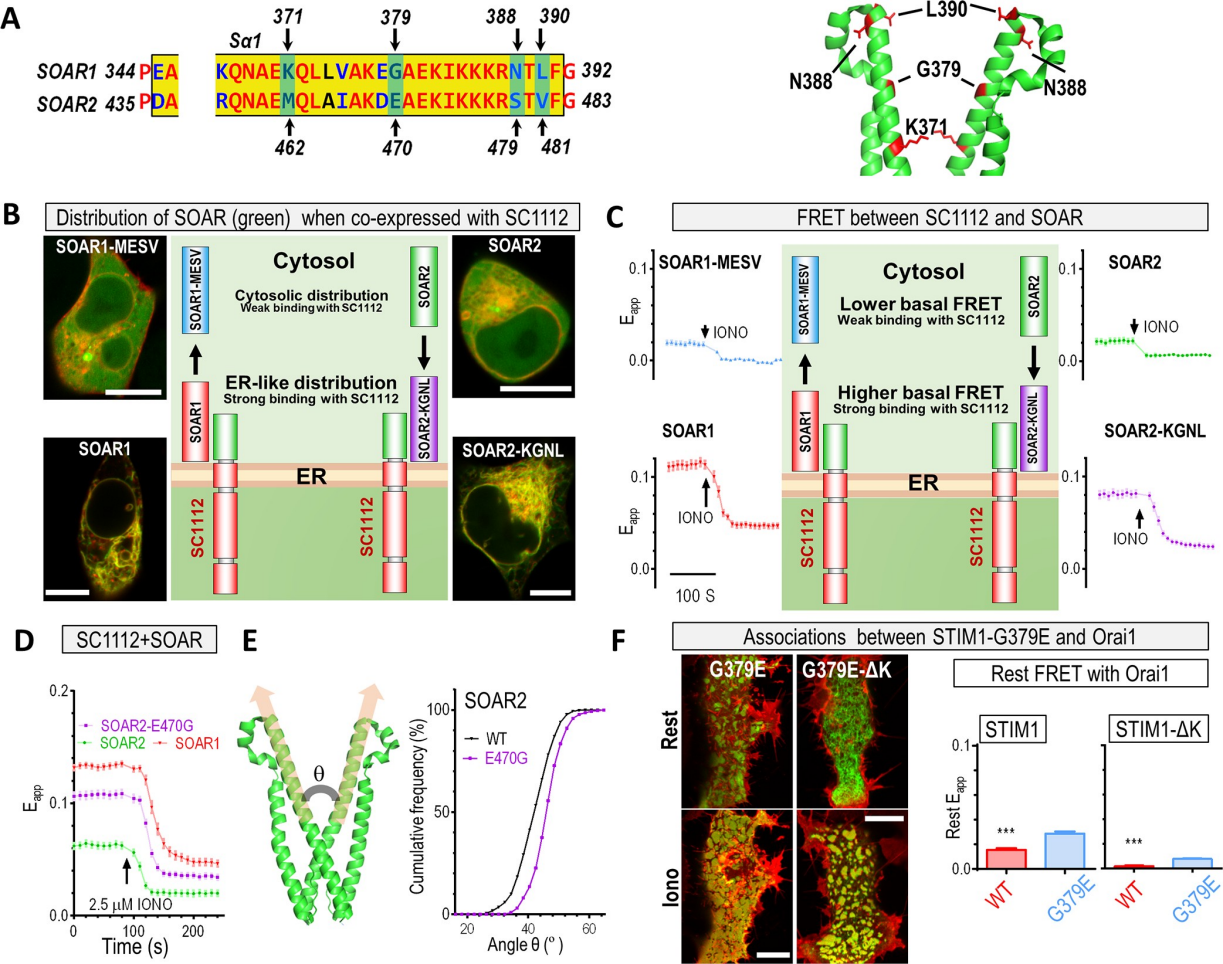
E470 residue in SOAR2 α1 region weakens its interactions with STIM_CC1_. (A) Four critical residues in the SOAR α1 region. Left: alignment of a partial sequence of the α1 region of SOAR1 and SOAR2, with the four critical residues indicated by arrows. Right: cartoon of a portion of a SOAR1 dimer crystal structure, with the four residues marked in red. (B–C) Comparative analysis of YFP-SOAR1, YFP-SOAR1-MESV, YFP-SOAR2, and YFP-SOAR2-KGNL constructs coexpressed with SC1112-CFP in HEK293 cells. (B) Typical confocal images of the distribution of the cytosolic SOAR constructs (green) and SC1112-CFP (red) (scale bar, 10 μm). Middle: cartoons illustrating the effects of substitutions on subcellular distributions of SOAR molecules coexpressed with SC1112-CFP (at least 12 cells for each condition were examined each time; *n* = 3). (C) Representative traces of FRET signals between SC1112-CFP and SOAR constructs. Middle: cartoons illustrating the effects of substitutions on FRET signal. (D) Typical traces showing the effect of E470G substitution on the FRET signals between SC1112-CFP and SOAR2. (E) Results of computer simulations demonstrating the effect of the E470G substitution on the angle between SOAR2 monomers. (F) FRET signals (bar graphs) and confocal images (left) demonstrating the associations or colocalization of Orai1 and STIM variants. Scale bar, 10 μm (*n* = 3, ***P < 0.001, *t* test). Individual numerical values underlying (C)–(F) may be found in S1 Data. CC1, coiled-coil 1; CFP, cyan fluorescent protein; ER, endoplasmic reticulum; FRET, Förster resonance energy transfer; HEK293, human embryonic kidney cells; IONO, ionomycin; KGNL, M462K-E470G-S479N-V481L; MESV, K371M-G379E-N388S-L390V;SC, STIM_1-CC1_ construct; SOAR, STIM-Orai–activating region; STIM, stromal interaction molecule; WT, wild type; YFP, yellow fluorescent protein.

Following this, we used site-directed mutagenesis to identify crucial residues within the SOAR α1 helix. The α1 helices of SOAR1 and SOAR2 differ by nine residues (Fig 4A, left). Mutagenesis studies revealed four residues that were crucial for CC1–SOAR interactions (Fig 4B). When these four residues in SOAR1 (K371, G379, N388, L390; KGNL) were substituted with those of STIM2 (M462, E470, S479, V481; MESV), the ability of the resulting SOAR1-K371M-G379E-N388S-L390V (SOAR1-MESV) variant to colocalize with a coexpressed SC1112 was greatly diminished, resulting in an even cytosolic distribution (Fig 4B, top left image versus bottom left image), with the construct behaving like SOAR2 (Fig 4B, top right image). Conversely, the introduction of the corresponding SOAR1 residues into SOAR2 (SOAR2- M462K-E470G-S479N-V481L, SOAR2-KGNL) rendered the SOAR2 variant SOAR1-like, with appreciable docking to the ER membrane and pronounced colocalization with the coexpressed SC1112 (Fig 4B, bottom right image versus bottom left image). This trend was further quantitatively confirmed by FRET assays: the basal FRET signals between SC1112 and SOAR constructs bearing MESV residues (Fig 4C, top two traces) were lower than those containing KGNL residues (Fig 4C, bottom two traces). Together, these experiments identified the four critical residues within SOAR that largely determine the relative strength of CC1–SOAR interaction locking the SOAR molecules in the vicinity of the ER membrane.

To pinpoint the residue that was most critical for the differential CC1–SOAR interactions of STIM1 and STIM2, we generated chimeric SOAR variants with single STIM1/STIM-swapping substitutions. When E470G was introduced into SOAR2, its FRET signal with SC1112 was substantially enhanced to a level that was comparable with the SC1112–SOAR1 interaction (Fig 4D). Conversely, introduction of G379E (a residue equivalent to STIM2-E470 within STIM1) into SOAR1 resulted in a significant reduction of the FRET signal (S5A Fig, right panel). To investigate the possible causes for this disruptive effect, we monitored the effect of SOAR2-E470 or SOAR1-G379 substitutions with different types of amino acid residues. As demonstrated, residues with side chains larger than that of alanine tended to result in a reduced basal FRET signal (S5A Fig). Collectively, these experiments indicated that the spatial constraints imposed by SOAR2-E470 might weaken the CC1–SOAR interaction.

Next, we addressed the question of why a single amino acid substitution had such a dramatic effect on STIM activation. We calculated the principal axis of each α1 helix within monomeric SOAR subunit and obtained the angle between the two α1 helixes (Fig 4E). This revealed that the angle of the SOAR2-E470G dimer was wider than that of SOAR2 (43.9° ± 0.1 ° versus 42.8° ± 0.1°, *P* < 0.0001, *t* test, *n* = 3), indicating that such a wider angle may facilitate the interactions between SOAR2 and the STIM2-CC1 domain. Consistent with this, the angle of the SOAR1-G379E dimer, which only weakly interacted with STIM1-CC1, was narrower than that of wild-type (WT) SOAR1 (36.6° ± 0.1° versus 38.9° ± 0.1°, *P* < 0.0001, *t* test, *n* = 3). Although the potential conformational change induced by these chimeric mutations was moderate, the shift in the size of angles after mutagenesis clearly demonstrated that the proper alignment of SOAR monomers is critical for the function of the SOAR dimer.

We then examined the effect of the weak CC1–SOAR interaction associated with SOAR2-E470 or SOAR1-G379E on the full-length STIM at rest. Since the low Ca^2+^ affinity of STIM2-EF-SAM rendered it partially active at rest and masked the actual basal cytosolic configuration of STIM2, we investigated the effect of the chimeric G379E substitution on the resting configuration of STIM1 cytosolic region. We reasoned that if the cytosolic region of STIM1-G379E was more extended than the WT, its C terminus would be closer to the PM, resulting in a higher basal FRET with PM-localized Orai1. The data agreed with this prediction (Fig 4F, bar graph on the left). Even after deletion of the PM-tethering K-rich region, the resulting STIM1-G379E-ΔK still has higher basal FRET signal with Orai1 (Fig 4F, bar graph on the right), again indicating a more extended configuration of the cytosolic region of STIM1-G379E. Moreover, STIM1-G379E, but not STIM1-G379E-ΔK, could form constitutive puncta under resting condition. This result thus revealed that the SOAR-bearing cytosolic region of STIM1-G379E variant was open enough to expose the membrane-anchored K-rich region to form constitutive puncta (Fig 4F, top images) [43, 50, 51]. We then checked whether the SOAR region in STIM1-G379E is close enough to the PM to engage and activate Orai1 channels. Confocal imaging results showed that neither STIM1-G379E nor STIM1-G379E-ΔK would colocalize with Orai1 channels at rest (Fig 3F, top two images). Thus, the cytosolic region of STIM1-G379E mutants were not extended enough to interact with Orai1 channels on PM. Consistent with this notion, STIM1-G379E WT or ΔK mutants did not induce constitutive Ca^2+^ entry (6.6 ± 1.8 nM and 3.5 ± 0.6 nM, respectively; *P* > 0.14 as compared with blank controls, *n* = 3). Overall, these findings clearly indicated that the chimeric STIM1-G379E adopted a more activated cytosolic configuration than WT STIM1, presenting the SOAR1 region closer to the PM at rest than WT STIM1 (Fig 4F).

Taken together, the data indicated that critical residues within SOAR2 define the start point of this transition, with SOAR2 located further away from the ER membrane and closer to the PM.

### E470 of STIM2 accounts for its weak coupling with Orai1

**Fig 5:**
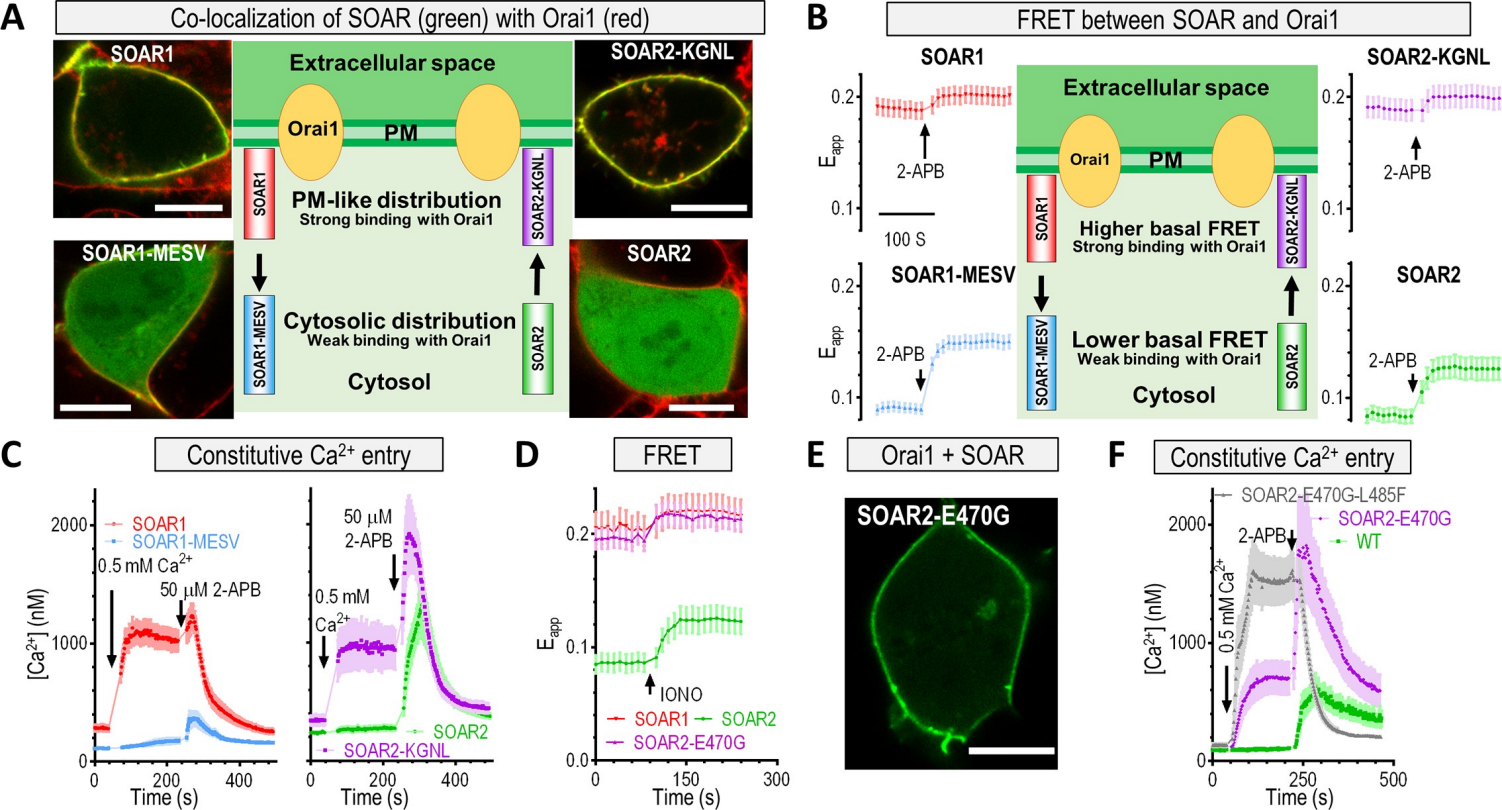
SOAR2-E470 determines the weak efficacy of SOAR2 to couple and activate Orai1 channels. (A–C) Comparative analysis of YFP-SOAR1, YFP-SOAR1-MESV, YFP-SOAR2, and YFP-SOAR2-KGNL constructs transiently expressed in HEK293-Orai1-CFP stable cells. A) Typical confocal images of the distribution of the cytosolic SOAR constructs and Orai1-CFP (scale bar, 10 μm). Middle: cartoons illustrating the effects of substitutions on the subcellular distribution of the SOAR molecules coexpressed with Orai1 (*n* = 3, at least 40 cells examined for each construct). (B) Representative traces of FRET signals between Orai1-CFP and SOAR constructs. Middle: cartoons illustrating the effects of substitutions on FRET signals. (C) Typical traces of a constitutive Ca^2+^ influx. (D–F) Analysis of SOAR2-E470G or SOAR2-E470G-L485F variants transiently expressed in HEK293-Orai1-CFP stable cells. (D) Representative traces of FRET signals between Orai1-CFP and SOAR2-E470G or WT SOAR. (E) Confocal images of the typical distribution of SOAR2-E470G molecules expressed in HEK293-Orai1-CFP stable cells (*n* = 3, at least 47 cells examined for each construct). Scale bar, 10 μm. (F) Typical traces of a constitutive Ca^2+^ influx mediated by WT SOAR or corresponding variants. Individual numerical values underlying (B–F) may be found in S1 Data. 2-APB, 2-Aminoethoxydiphenyl borate; CFP, cyan fluorescent protein; FRET, Förster resonance energy transfer; HEK293, human embryonic kidney 293 cells; IONO, ionomycin; KGNL, M462K-E470G-S479N-V481L; MESV, K371M-G379E-N388S-L390V; PM, plasma membrane; SOAR, STIM-Orai–activating region; STIM, stromal interaction molecule; WT, wild type; YFP, yellow fluorescent protein.

Upon activation, the cytosolic region of STIM overcomes the CC1-SOAR–mediated autoinhibition to expose SOAR to the PM, engage, and activate Orai1 channels [38, 44, 45]. We previously identified a residue within the SOAR α2 region, STIM1-F394 or STIM2-L485, that defines the distinct Orai1-activating ability of STIM1 and STIM2 [37]. Even though the SOAR1-F394H mutant lost its ability to bind with Orai1 [37, 52], the chimeric mutations, SOAR1-F394L or SOAR2-L485F, still retain the same Orai1-binding abilities as the corresponding WT SOAR molecules, indicating a minimal role of SOAR1-F394 or SOAR2-L485 for Orai1 binding. What defines the distinct Orai1-binding behavior of SOAR1 and SOAR2 still remains elusive. We asked whether the identified four amino acid residues also impacted the SOAR–Orai1 interaction, i.e., the late step of the intermolecular switch, since they are located in close proximity to five positively charged residues (^382^KIKKKR^387^) that are essential for the binding of SOAR to Orai1 [44, 53]. Upon coexpression with Orai1, the SOAR1-MESV variant exhibited a SOAR2-like cytosolic distribution (Fig 5A, bottom left image), indicating a weak interaction with Orai1. By comparison, the SOAR2-KGNL variant was SOAR1-like, with clear PM decoration, suggesting a strong interaction with Orai1 (Fig 5A, top right image). This tendency was quantitively confirmed by FRET imaging (Fig 5B). Functionally, MESV substitutions in SOAR1 almost abolished its ability to induce constitutive Ca^2+^ influx, while the SOAR2-KGNL variant acquired an Orai1-activating ability that was similar to SOAR1 (Fig 5C). Pharmacologically, all these constitutively Ca^2+^ influxes have corresponding typical 2-APB responses mediated by SOAR2-Orai1 or SOAR1-Orai1 [37], indicating that these Ca^2+^ entries are mostly mediated by Orai1 channels. Together, these findings indicated that the four critical residues determined not only the SOAR’s distinct capability to interact with CC1 at rest but also the ability to engage and activate the Orai1 channel.

We next examined whether SOAR2-E470 was crucial for the interaction with Orai1. In cells stably expressing Orai1-CFP, the basal FRET signal between Orai1 and SOAR2-E470G was significantly stronger than that of SOAR2 and similar to that of SOAR1 (Fig 5D). Accordingly, the chimeric E470G substitution resulted in an altered cellular distribution of SOAR2, from dispersed cytosolic to mostly PM associated, echoing the behavior of SOAR1 (Fig 5E). This indicated that the SOAR2-E470G protein was able to couple with Orai1 with a similar efficacy as SOAR1. Indeed, the SOAR2-E470G variant induced constitutive Ca^2+^ entry on a scale close to that of SOAR1 (Fig 5F). After the introduction of an additional substitution—L485F, previously identified to enhance the gating of Orai1 [37]—the resulting double-variant SOAR2-E470G-L485F generated an even larger constitutive Ca^2+^ influx, fully recapitulating the function of SOAR1 (Fig 5F). The behavior of the corresponding SOAR1 chimeric variants was consistent with these observations (S5B Fig, right panel). Taken together, our findings demonstrated that the E470 residue of SOAR2 defines the protein’s distinct efficacy of Orai1 binding, leading to weaker activation of Orai1 channels upon STIM2 activation.

**Fig 6:**
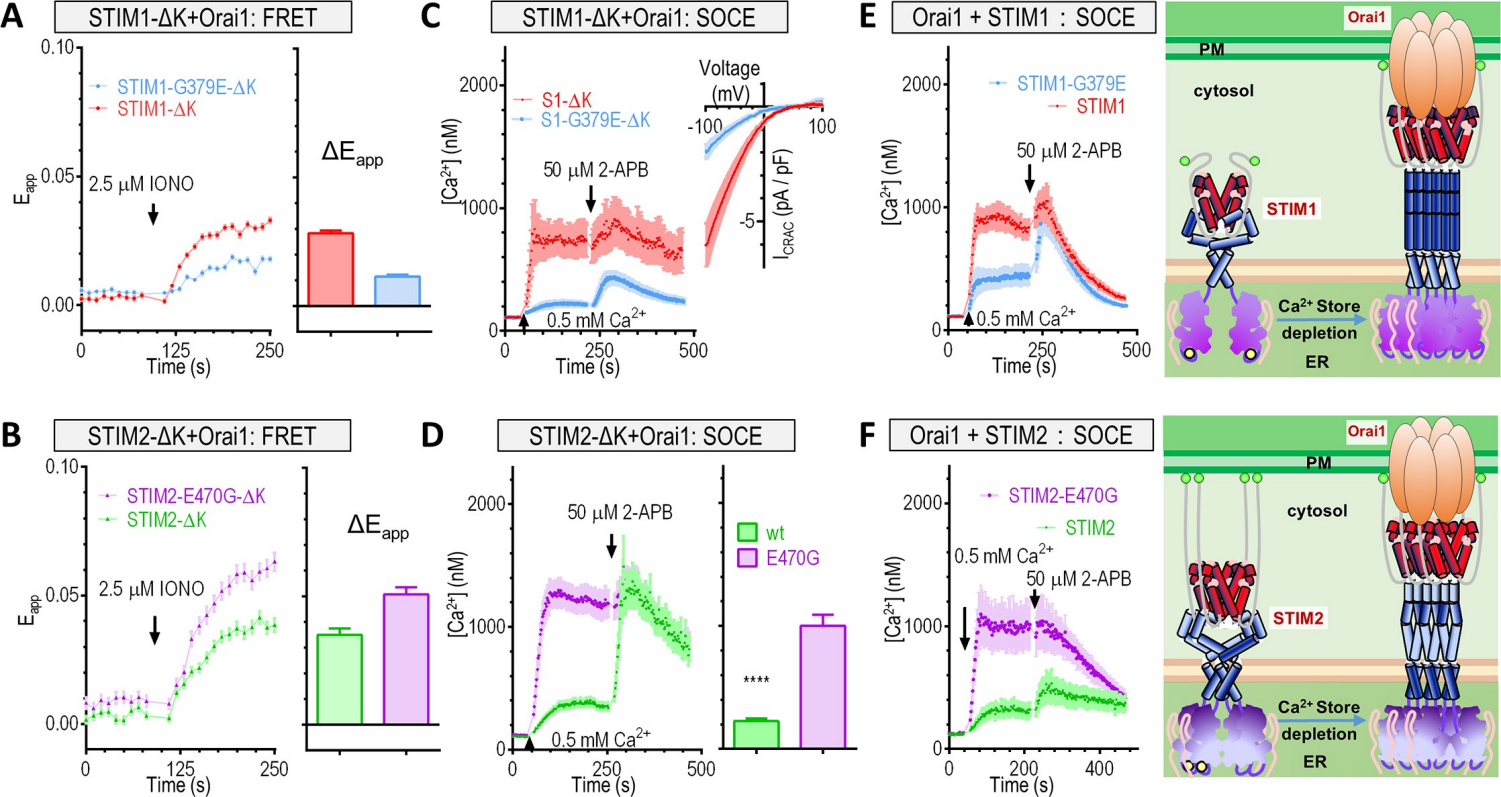
STIM2-E470 restricts STIM2 activation and its ability to induce SOCE. In HEK293 Orai1-CFP stable cells, the effects of G379E or E470G substitutions on the coupling of transiently expressed STIM or STIM-ΔK with Orai1 were examined. (A–B) Rest and IONO-induced FRET signals between Orai1 and (A) STIM1 or STIM1-G379E or (B) STIM2 or STIM2-E470G. Each left: typical traces; each right: statistical analysis. (C–D) SOCE responses. (C) Typical SOCE (left) or average I_CRAC_ (right) responses for STIM1-ΔK and STIM1-G379E-ΔK. For Ca^2+^ imaging traces (left), the ER Ca^2+^ store was emptied by a 10 min incubation in 1 μM TG before the recordings (*n* = 3). I–V relationships at the peak of whole cell current are also shown (right, *n* = 6). (D) SOCE responses for STIM2-ΔK and STIM2-E470G-ΔK. Left, typical traces; right, statistical analysis (****P < 0.0001, *t* test, *n* = 3). (E–F) Typical traces showing the effects of STIM1-G379E or STIM2-E470G substitutions on SOCE responses mediated by Orai1 and full-length STIM. Diagrams on the right: A model illustrating distinct SOCE activation modes mediated by STIMs (please refer to S5C Fig for a simplified version). Upon store depletion, similar to STIM1 (top panel), the cytosolic region of STIM2 (bottom panel) also undergoes conformational changes to further engage and activate Orai1 channels. The activation of STIM2 has several distinct features. First, the relatively lower Ca^2+^ affinity of STIM2-EF-SAM keeps STIM2 partially and constitutively active. Second, the SOAR2-E470 narrows down activation dynamics of STIM2 with two effects: At rest, it impairs the efficient caging by CC1, presenting SOAR2 close to the PM. Upon activation, it restricts the Orai1-binding efficacy of SOAR, keeping SOAR2 from getting too close to Orai1 on the PM. Thus, the SOAR2 region of STIM2 only needs to move a smaller distance to switch STIM2 from a store-replete mode to a store-depleted one, leading to a faster STIM2-mediated SOCE process so that partially emptied ER Ca^2+^ stores can be efficiently refilled via STIM2-activated SOCE. Individual numerical values underlying (A)–(F) may be found in S1 Data. 2-APB, 2-Aminoethoxydiphenyl borate; CC1, coiled-coil 1; CFP, cyan fluorescent protein; CRAC, Ca^2+^-release–activated Ca^2+^ current; EF-SAM, EF-hand and sterile alpha motif domain; ER, endoplasmic reticulum; FRET, Förster resonance energy transfer; HEK293, human embryonic kidney 293 cells; IONO, ionomycin; I–V, current–voltage; PM, plasma membrane; SAM, sterile alpha motif; SOAR, STIM-Orai–activating region; SOCE, store-operated Ca^2+^ entry; STIM, stromal interaction molecule; TG, thapsigargin; WT, wild type.

We then examined the effect of the chimeric substitutions in STIM-ΔK or full-length STIM on the coupling and subsequent activation of Orai1. Similar to results obtained from SOAR fragments, G379E mutation significantly decreased the maximal FRET signals between store-activated STIM1-ΔK and Orai1 (Fig 6A). Conversely, STIM2-E470G-ΔK and Orai1 produced larger FRET signals than STIM2 (Fig 6B). Both results revealed that E470/G379 critically defines the coupling of Orai1 with STIM lacking its PM-anchoring K domain, or STIM-ΔK. Consequently, as anticipated based on the observations with SOAR variants (Figs 5F and S5B), the amplitude of I_CRAC_ and SOCE of the chimeric STIM1-G379E-ΔK variant were smaller than those of STIM1-ΔK in HEK293-Orai1 stable cells (Fig 6C). In contrast, the chimeric STIM2-E470G-ΔK variant induced a substantially larger SOCE than WT STIM2-ΔK (Fig 6D), reaching a level comparable to STIM1-ΔK. Chimeric substitution in full-length STIM expressed similar effects in HEK293-Orai1 stable cells: the STIM1-G379E variant behaved like STIM2, inducing smaller SOCE than STIM1 (Fig 6E); and STIM2-E470G functioned similarly to STIM1, mediating larger SOCE than STIM2 (Fig 6F). Taken together, these results showed that E470 of STIM2 defines its weaker capability to couple and activate Orai1.

Overall, our results indicate that STIM2-E470 critically narrows down the dynamic rearrangement of the SOAR2 region during STIM2 activation by defining the lesser efficacy of SOAR2 to interact with CC1 at rest (Fig 5D) and to couple with Orai1 once activated (Figs 6A and 6B and S5C; Fig 6, diagrams on the right). Thus, the corresponding G379E substitution would present the SOAR1 region in STIM1 closer to the PM at rest than WT STIM1-ΔK (Fig 4F), then STIM1-G379E-ΔK would undergo a relatively smaller rearrangement to engage and activate Orai1 after activation. Indeed, compared with WT STIM1-ΔK, Ca^2+^ store depletion after the addition of ionomycin induced a reduced FRET increase between STIM1-G379E mutant and Orai1, indicating impaired dynamics of its cytosolic region (Fig 6A). In contrast, compared with WT STIM2, the cytosolic region of STIM2-E470G-ΔK mutant underwent more pronounced dynamic changes and induced greater FRET changes (Fig 6B). Collectively, the data indicated that the E470 residue of STIM2-ΔK enabled a more open cytosolic confirmation at rest, resulting in smaller activation dynamics (diagrams in Figs 6 and S5C).

In summary, we used FRET-based biosensor to systemically dissect the mechanism of STIM2 activation. Together with previously reported findings, observations made in the current study enable us to propose a model that could reconcile the paradox between the distinct mode of STIM2 activation and the previously proposed STIM2 physiological functions. Briefly, its low Ca^2+^ affinity renders STIM2 partially active at rest, constitutively inducing Ca^2+^ entry via STIM2-activated Orai1 channels. The protein regions upstream of the SOAR2 domain ensure a slow activating kinetics, while the E470 and L485 residues within SOAR2 are responsible for the weak engagement and activation of Orai1 by STIM2. These two factors constrain STIM2-mediated Ca^2+^ entry, thus preventing potential Ca^2+^ overload associated with the constitutively active STIM2. The low Ca^2+^ affinity also renders STIM2 sensitive to small fluctuations in the ER Ca^2+^ levels and enables a partially activated configuration of the STIM2 cytosolic region. The weak docking of SOAR2 to CC1 further unfolds the cytosolic STIM2 region, placing the Orai1-activating SOAR2 region in the vicinity of Orai1 at rest. Once further activated by a small reduction of the ER Ca^2+^ levels, SOAR2 only has to move a relatively short distance to engage with and activate Orai1 channels. Thus, the small dynamic range of STIM2 activation compensates for the slow activating kinetics of STIM2 and might ensure a rapid response to the fluctuations in the ER Ca^2+^ levels, rendering it an efficient regulator for the maintenance of ER Ca^2+^ homeostasis [23].

## Materials and methods

### Plasmid construction

To generate full-length STIM1 and STIM2 constructs in pECFP-N1 and pEYFP-N1 vectors (Clontech, Mountain View, CA, USA), STIM1 and STIM2 were amplified from STIM1-YFP in MO91 vector [54] and STIM2-YFP in pIRESneo vector [32], respectively; they were inserted into pECFP-N1 and pEYFP-N1, respectively, between the Xhol and BamHI sites. To generate chimeric STIM constructs in pECFP-N1 or pEYFP-N1 vectors (shown in S1 Fig), various STIM fragments and vector fragments were amplified from STIM-YFP or STIM-CFP (in pECFP-N1 or pEYFP-N1) and ligated using the NEBuilder HiFi DNA assembly enzyme (New England BioLabs, Ipswich, MA, USA). To construct chimeric STIM_N-EF-SAM-TM-CC1_-CFP capable of translocation from the ER to the PM, the Myc tag was first inserted between SP and EF-SAM to track chimeric STIM location. Then, the ER-targeting SP of STIM was replaced by the extracellularly targeting SP from CD8A_1-21_. Next, the trafficking SP from Kir2.1_233−252_ and ER-exporting SP from Kir2.1_374−380_ were inserted upstream and downstream of the CFP tag, respectively, using standard PCR and T4 ligation. mCh-CAD was purchased from Addgene (#73566; Cambridge, MA, USA). YFP-SOAR was generated as previously described [8]. YFP-SOAR1L (STIM1_343-491_) and YFP-SOAR2 (STIM2_435-533_) were generated by replacing the SOAR1-coding sequence in YFP-SOAR with that of SOAR1L or SOAR2. To generate a chimeric YFP-SOAR construct from SOAR1 and SOAR2, the SOAR fragments and vector fragments were PCR amplified and then ligated by using the NEBuilder HiFi DNA assembly enzyme (New England BioLabs). The corresponding variants of all STIM constructs were generated using the QuikChange Lightning multisite-directed mutagenesis kit (Agilent, Santa Clara, CA, USA).

### Cell culture and transfection

HEK293 and HeLa cells were cultured in DMEM (HyClone, Chicago, IL, USA) containing 10% FBS (cat: 900–108, Gemini Bio-Products, West Sacramento, CA, USA) and 5% penicillin and streptomycin (Thermo Scientific, Waltham, MA, USA) at 37°C with 5% CO_2_ [55]. Transfections were performed by electroporation using the Bio-Rad Gene Pulser Xcell system (Bio-Rad, Hercules, CA, USA) in 4 mm cuvettes and OPTI-MEM medium [55, 56]. For HEK293 cells, a voltage step pulse (180 V, 25 ms, in 0.4 ml of the medium) was used; for HeLa cells, an exponential pulse (260 V, 525 μF, in 0.5 ml medium) was used. After electroporation, the cells were seeded on round coverslips and cultured in OPTI-MEM medium for another 45 min before FBS was added to a final concentration of 7%. All experiments were carried out 24 h after transfection.

### Fluorescence imaging

Fluorescence imaging of time-series experiments was conducted using a ZEISS observer Z1 microscope equipped with X-Cite 120-Q (Lumen Dynamics, Waltham, MA, USA) light source, 40× oil objective (NA 1.3), and Axiocam 506 mono Camera (Zeiss, Oberkochen, Germany). The imaging system was controlled with the Zen software. All filters or filter sets were purchased from Semrock (BrightLine; Semrock, Rochester, NY, USA). Both FRET and Ca^2+^ imaging were performed using this system at room temperature (20°C), as previously described [47, 55]. Data were acquired from cells that had been cultured on round coverslips placed in the imaging solution. The imaging solution contained 107 mM NaCl, 7.2 mM KCl, 1.2 mM MgCl_2_, 11.5 mM glucose, and 20 mM HEPES-NaOH (pH 7.2). For single-cell intracellular cytosolic Ca^2+^ measurements, the cells were first bathed in the imaging solution containing Fura-2 AM for 1 h to get Fura-2 AM loaded into cells and then de-esterified. The cytosolic Ca^2+^ signals were then acquired using a FURA2-C-000 filter set. Emission fluorescence signal at 510 ± 42 nm generated by light at the 340 ± 12.5 nm excitation wavelength (F_340_) and at 387 ± 5.5 nm (F_380_) was acquired every 2 s; the intracellular Ca^2+^ levels are collected as F_340_/F_380_ ratio [57]. For FRET measurements, CFP (438 ± 12 nm_Ex_/483 ± 16 nm_Em_), YFP (500 ± 12 nm_Ex_/542 ± 13.5 nm_Em_), and FRET_raw_ (438 ± 12 nm_Ex_/542 ± 13.5 nm_Em_) filters were used for image acquisition (F_CFP_, F_YFP_, and F_raw_, respectively) every 10 s at room temperature. The corresponding fluorescence readings from regions of interest were exported from the Zen software and imported into Matlab 2014a (The MathWorks, Natick, MA, USA) to calculate the F_340_/F_380_ ratio, or the system-independent apparent FRET efficiency, E_app_ [55, 58, 59]. The parameters and calculation methods used to generated E_app_ values from raw fluorescent signals were the same as those previously described [47]. The calibration of Fura-2 signals was done with a modified protocol based on a previously described one [60]. Fura-2 traces in 10 mM EGTA or 30 mM Ca^2+^ were fitted with corresponding exponential equations to achieve more accurate maximal or minimal Fura-2 signals. The resulting traces or values of Ca^2+^ concentrations or E_app_ were plotted using the Prism 7 software. Representative traces from at least three independent experiments are shown as mean ± SEM.

In situ Ca^2+^ titration of R-CEPIA1er and measurements of ER Ca^2+^ levels with R-CEPIA1er were performed as the following. HeLa WT or SK cells transiently coexpressing R-CEPIA1er and a Ca^2+^-insensitive ER marker, CFP-Sec61β, were used for measurements. CFP (438 ± 12 nm_Ex_/483 ± 16 nm_Em_) or a TxRed-A-Basic-000 filter was used to collect the corresponding CFP or R-CEPIA1er fluorescence every 2 s. In situ calibration of CEPIA1er signals was performed in a calibration solution containing 10 mM NaCl, 140 mM KCl, 1 mM MgCl_2_, 20 mM HEPES, 0.025 mM digitonin, and 0.01 mM ionomycin (pH 7.2) [26, 61]. To avoid artifacts caused by cell movements and leaking of R-CEPIA1er after cell permeabilization, the fluorescence ratio between R-CEPIA1er and CFP-sec61β (R) were used for calibration. During calibration, cells were first permeabilized with the above solution containing various amounts of Ca^2+^ for 4 min to obtain the response ratio (R), and the response to 30 mM CaCl_2_ was taken as the maximum response (R_max_). Then, Ca^2+^ was removed and 1 mM EGTA was added in the bathing solution to obtain the minimum response (R_min_). The in situ apparent Ca^2+^ affinity (K_d_ = 467 ± 20 μM) and Hill coefficient (Hill_n_ = 1.46 ± 0.08) of R-CEPIA1er were then calculated through curve fittings with the following equation [26]:

$${\left[\text{C}{\text{a}}^{2+}\right]}_{\text{free}}={\text{K}}_{\text{d}}\cdot {\left[(\text{R}–{\text{R}}_{\text{min}})/\left({\text{R}}_{\text{max}}-\text{R}\right)\right]}^{1/\text{n}}$$

Afterwards, the ER Ca^2+^ levels were calculated using the following equation [26]:

$${\left[\text{C}{\text{a}}^{2+}\right]}_{\text{ER}}={\text{K}}_{\text{d}}\cdot {\left[(\text{R}–{\text{R}}_{\text{min}})/\left({\text{R}}_{\text{max}}-\text{R}\right)\right]}^{1/\text{n}}$$

The obtained in situ values of CEPIA1er indicators were used in further calculations [26].

All experiments were carried out at room temperature. Traces shown are representative of at least three independent repeats, with 15–60 single cells analyzed per each repeat.

### In situ calibration of Ca^2+^ affinities of STIM constructs

The FRET signals between the YFP-SOAR1L-SC1111/1121 pairs and R-CEPIA1er signals were acquired simultaneously from HEK293 cells or HeLa SK cells transiently coexpressing R-CEPIA1er, YFP-SOARL, and SC1111-CFP or SC1211-CFP. The same filters as described in the above sections were used. For measurements of Ca^2+^ affinities of full-length STIM1 using STIM1 puncta as readouts, confocal microscopy (described below) was used to collect corresponding YFP and R-CEPIA1er signals. ER Ca^2+^ stores were gradually depleted with 1 μM thapsigargin (TG); the resulting decrease in FRET signal or increases in punctate area were then plotted against the corresponding ER Ca^2+^ levels indicated by R-CEPIA1er fluorescence. Similar to previous reports [23, 30], either densities of STIM puncta or the FRET signals between YFP-SOAR1L and SC1111-CFP or SC1211-CFP functioned as an indicator of STIM activation in the current study. Ca^2+^ affinities of the STIM constructs were then calculated by fitting the obtained puncta/FRET-Ca^2+^ relationship to the Hill equation using Prism 7 software.

### Confocal microscopy

Subcellular distribution of fluorescently tagged STIM and Orai1 constructs was monitored using a Zeiss LSM 880 confocal system equipped with a 100× oil lens (NA 1.45; Zeiss). The acquired raw images were analyzed using Image J software (NIH). Studies of the PM targeting of STIM- and STIM-CC1–binding SOAR were conducted using the Nikon Eclipse Ti-E microscope (Nikon Instruments, Tokyo, Japan) equipped with an A1R-A1 confocal module with LU-N4 laser sources and CFI Plan Apochromat VC series Objective Lenses (60× or 40×). All acquired confocal images were analyzed by using the NIS-Elements AR microscope imaging software (Nikon, NIS-element AR version 4.0).

### Live-cell immunofluorescence staining

To confirm the orientation of either end of the engineered PM-STIM protein, live-cell immunofluorescence staining without cell permeabilization was performed. HeLa cells were grown on 35 mm glass-bottomed dishes (MatTek, Ashland, MA, USA) and transfected with PM-STIM constructs using Lipofectamine 3000 [62]. Next, 18 h post-transfection, cells were incubated with c-Myc antibody (1:400 dilution; Santa Cruz Biotechnology Cat#: sc-41; Santa Cruz Biotechnology, Dallas, TX, USA) in regular DMEM medium at 37°C incubator and under 5% CO_2_ for 1 h. The cells were then washed twice with fresh culture medium. Secondary goat anti-mouse IgG Alexa Fluor 568 antibody (1:500 dilution; Z25106, Thermo Fisher) was then incubated with the cells in the growth medium for 1 h. After extensive washing, images were acquired using a Nikon A1R confocal microscope at 40× magnification.

### Electrophysiological measurements

Data were collected using an HEKA EPC 10 USB double patch amplifier controlled by Patchmaster software (HEKA Elektronik, Lambrecht/Pfalz, Germany). The I_CRAC_ in HEK293 Orai1-CFP stable cells transiently expressing STIM1-ΔK-YFP or corresponding 1121 or G379E variants was measured with conventional whole-cell recordings [38]. After the establishment of the whole-cell configuration, a holding potential of 0 mV was applied. A 50 ms step to –100 mV followed by a 50 ms ramp from –100 to +100 mV was delivered every 2 s. Currents were low-pass–filtered at 2.3 kHz (four-pole Bessell) and acquired at a sampling rate of 10 kHz. HEKA Fitmaster and Matlab 2014b software were used for offline data analysis, and the currents were further low-pass–filtered at 500 Hz. The intracellular or pipette solution contained 135 mM Cs-aspartate, 8 mM MgCl_2_, 10 mM EGTA, and 10 mM Cs-HEPES (pH 7.2). The extracellular solution contained 130 mM NaCl, 4.5 mM KCl, 20 mM CaCl_2_, 10 mM TEA-Cl, 10 mM d-glucose, and 5 mM Na-HEPES (pH 7.4). A 10 mV junction potential compensation was applied to correct the liquid junction potential of the pipette solution relative to the extracellular solution. Currents from at least six cells for each condition were collected and averaged.

### Computer simulations

To determine the dynamics of SOAR1, its initial dimeric structure was taken from the X-ray structure (PDB: 3TEQ). The other structures (SOAR2, SOAR1-G379E, SOAR1-MESV, SOAR2-E470G, and SOAR2-KGNL) were built by homology modeling with SOAR1 as the template; the modeling was conducted using the Swiss-model server [63, 64]. For each construct, the protein molecule was solvated in a cubic water box. To mimic the physiological environment, 150 mM NaCl was introduced; extra Cl^-^ ions were introduced to neutralize the system. The total number of atoms was approximately 84,000. The CHARMM 36 force field [65] was used for protein and ions, and the TIP3P model was used for water [66].

Molecular dynamics (MD) simulations were performed using the NAMD package suite [67]. The simulations were run at constant temperature (300 K) and constant pressure (1 bar). The particle mesh Ewald method [68] was used to treat long-range electrostatic interactions, with a cutoff of 12 Å. Integration time step was set at 2 fs, with all bonds containing hydrogen held rigid. Langevin dynamics were used to control the temperature, while the pressure of the system was controlled by the Nosé-Hoover-Langevin piston [69]. Each system was accumulated over a 400 ns trajectory, and data from the last 200 ns were used for analysis.

## Supporting information

S1 Fig Overview of the engineered STIM constructs (chimeras or mutants). (A–B) Full-length STIM chimeras or constructs with C-terminal deletions. (C–D) SCs and chimeras; (E–F) PM-anchoring SCs and chimeras; (G–H) SOAR constructs and chimeras. C, CFP tag; CC1, coiled-coil 1; CFP, cyan fluorescent protein; EF-SAM, EF-hand and sterile alpha motif domain; K, lysine-rich region; NT, N terminus; PM, plasma membrane; SC, STIM_1-CC1_ construct; SOAR, STIM-Orai–activating region; SOAR1L, STIM1(343–491); STIM, stromal interaction molecule; sα1–4: α helix 1 to 4 of SOAR region; TM, transmembrane region; Y, YFP tag; YFP, yellow fluorescent protein.
(TIF)

S2 Fig Engineering strategies used to enable ER-to-PM trafficking of STIMs. (A) Schematic illustration of the strategies used to force the ER-to-PM trafficking of engineered STIMs. First, a Myc tag was introduced into STIM between SP and EF-SAM to aid the determination of the orientation of the N terminus of STIM. The original ER SP of STIM was replaced by an extracellularly targeting peptide derived from CD8A_1-21_. To facilitate the ER export of STIM1 and trafficking in the cytosol, PM-trafficking TP (Kir2.1_233−252_) and ER-exporting TP (Kir2.1_374−380_) were inserted upstream and downstream of the C-terminal CFP, YFP, or mCh fluorescent tag, respectively. (B) Live-cell immunofluorescence staining of HeLa cells expressing the designed YFP-tagged PM-targeting constructs. Alexa-Fluor-568–conjugated secondary antibody was used to determine the extracellular localization of the Myc tag in nonpermeabilized HeLa cells. (C) Confocal imaging of HeLa cells coexpressing PM-S2222-YFP and mCh-CAD cultured in the 2 mM Ca^2+^ medium or Ca^2+^-free medium. Scale bar, 10 μm. CAD, CRAC-activating domain; CFP, cyan fluorescent protein; CRAC, Ca^2+^-release-activated Ca^2+^ current; EF-SAM, EF-hand and sterile alpha motif domain; ER, endoplasmic reticulum; mCh, mCherry PM, plasma membrane; SP, signal peptide; STIM, stromal interaction molecule; TP, target peptide; YFP, yellow fluorescent protein.
(TIF)

S3 Fig Ca^2+^ affinities of various SCs. (A) In HEK293-Orai1 stable cells transiently expressing WT STIM or corresponding STIM chimeras with swapped EF-SAM regions, only cells expressing constructs that contain the STIM2 EF-SAM (STIM1211 or STIM2) facilitate a high constitutive Ca^2+^ influx (blue and green traces); no such constitutive Ca^2+^ influx was observed in cells expressing constructs harboring the STIM1 EF-SAM (red and purple traces). (B) Statistics showing Ca^2+^ affinity (mM) of the various PM-anchoring SCs. (C) Some unengineered SCs show some PM-like distribution in approximately 25% of transfected cells. FRET signals between YFP-SOAR1L and PM-localized SC-CFP constructs in response to increases in extracellular Ca^2+^ concentration in these cells. Left, typical traces; right, statistical analysis of the apparent K_d_ (*n* = 5, *P* = 0.0002). (D) Calibration of the ER Ca^2+^ levels using R-CEPIA1er and a Ca^2+^-insensitive ER marker, CFP-Sec61β in HeLa SK cells. Left, a typical trace used for calibration; right, statistics of the ER Ca^2+^ concentration. (E) In HeLa SK cells coexpressing R-CEPIA1er, YFP-SOAR1L, and SC1111-CFP or SC1211-CFP, ER Ca^2+^ levels and FRET signals between SCs and SOARL were monitored simultaneously. Typical traces of the rest state and TG-induced responses for R-CEPIA1er signals. Individual numerical values underlying (A)–(E) may be found in S1 Data. CFP, cyan fluorescent protein; EF-SAM, EF-hand and sterile alpha motif domain; ER, endoplasmic reticulum; FRET, Förster resonance energy transfer; HEK293, human embryonic kidney 293 cells; PM, plasma membrane; SC, STIM_1-CC1_ construct; SK, STIM1 and STIM 2 double knockout; SOAR, STIM-Orai–activating region; STIM, stromal interaction molecule; TG, thapsigargin; WT, wild type; YFP, yellow fluorescent protein.
(TIF)

S4 Fig FRET signals between SC and SOAR correlate well with the activation status of full-length STIMs. Panels with light yellow background are cells expressing constructs containing the STIM1 cytosolic region; panels with light cyan background are cells expressing molecules containing the STIM2 cytosolic region. (A–D) Comparison of the function of STIM1-YFP (A), STIM2-YFP (B), and the luminal-region–exchanged chimeras, STIM1122-CFP (C) or STIM2211-CFP (D), expressed in HEK293-Orai1-CFP cells or coexpressed with Orai1-YFP in HEK293 WT cells. Left, a diagram of the two coexpressed SOCE components. Top panel: confocal images of the typical cellular distribution of STIM1, STIM2, STIM1122, and STIM2211 at rest (scale bar, 10 μm). Bottom panel: representative traces for a constitutive Ca^2+^ entry into the Orai1- and STIM-coexpressing cells. (E–G) Comparative analysis of interactions between STIM_1-CC1_-CFP and YFP-SOAR molecules coexpressed in HEK293 tsA cells, the tsA201 variant of HEK293 cells expressing a temperature sensitive mutant of the SV40 large T antigen. (E) SC1111-CFP+YFP-SOAR1, (F) SC2222-CFP+YFP-SOAR2, (G) SC1122-CFP+YFP-SOAR2, and (H) SC2211-CFP+YFP-SOAR1. The top diagrams show the two coexpressed STIM fragments. Top panel: representative traces of typical FRET signals between WT or chimeric STIM_1-CC1_-CFP and YFP-SOAR molecules; Bottom panel: confocal images of the typical colocalization of STIM_1-CC1_-CFP and YFP-SOAR molecules (scale bar, 10 μm). All results are typical of at least three independent repeats, and at least 36 cells were examined for each condition. Individual numerical values underlying (A)–(H) may be found in S1 Data. CC1, coiled-coil 1; CFP, cyan fluorescent protein; FRET, Förster resonance energy transfer; HEK293, human embryonic kidney 293 cells; SC, STIM_1-CC1_ construct; SOAR, STIM-Orai–activating region; SOCE, store-operated Ca^2+^ entry; STIM, stromal interaction molecule; tsA, the tsA201 variant of HEK293 cells expressing a temperature sensitive mutant of the SV40 large T antigen; WT, wild type; YFP, yellow fluorescent protein.
(TIF)

S5 Fig Effects of SOAR1-G379 or SOAR2-E470 mutations on their associations with CC1 or functional coupling with Orai1. (A) Statistics showing resting FRET signals of SOAR constructs with SC1112 (left) or SC1111 (right). (***P < 0.001; *P < 0.05, *t* test, *n* = 3). (B) Statistics of mean constitutive Ca^2+^ influxes mediated by Orai1 and different SOAR2 (left) or SOAR1 (right) constructs. (***P < 0.001; *P < 0.05, *t* test, *n* = 3). Note: all signals were compared with and normalized to those mediated by SOAR1. Individual numerical values underlying (A) and (B) may be found in S1 Data. (C) A simplified, hypothetical cartoon showing how the binding of the SOAR within full-length STIM constructs with its CC1 or Orai1 would define the dynamic range of STIM activation. For simplicity, only CC1 and SOAR domains of the cytosolic region of STIMs were shown. The range of dynamic changes is indicated by the sizes of blue arrows. At rest, CC1 docks SOAR near the ER membrane, preventing it from activating Orai1 on the PM (bottom); after Ca^2+^ store depletion, SOAR detaches from CC1 and relocates to the vicinity of the PM, where it binds and activates the Orai1 channels. CC1, coiled-coil 1; ER, endoplasmic reticulum; FRET, Förster resonance energy transfer; PM, plasma membrane; SC, STIM_1-CC1_ construct; SOAR, STIM-Orai–activating region; STIM, stromal interaction molecule.
(TIF)

S1 Data Data underlying the study. (XLSX)

## Abbreviations

2-APB: 2-Aminoethoxydiphenyl borate
CAD: CRAC-activating domain
CC1: coiled-coil 1
CFP: cyan fluorescent protein
CRAC: Ca^2+^-release–activated Ca^2+^ current
E_app_: apparent FRET efficiency
EF-SAM: EF-hand and sterile alpha motif domain
ER: endoplasmic reticulum
FRET: Förster resonance energy transfer
GFP: green fluorescent protein
HEK293: human embryonic kidney 293 cells
IONO: ionomycin
I–V: current–voltage
mCh: mCherry
MD: molecular dynamics
ns: not significant
NT: N terminus
PM: plasma membrane
R: response ratio
SAM: sterile alpha motif
SC: STIM_1-CC1_ construct
SK: STIM1 and STIM2 double knockout
SOAR: STIM-Orai–activating region
SOCE: store-operated Ca^2+^ entry
SP: signal peptide
STIM: stromal interaction molecule
STIM-KGNL: K371-G379-N388-L390 residues or corresponding M462K-E470G-S479N-V481L mutant of STIM2 constructs
STIM-MESV: STIM2-M462-E470-S479-V481 residues or corresponding K371M-G379E-N388S-L390V mutants of STIM1 constructs
TG: thapsigargin
TM: transmembrane region
TP: target peptide
WT: wild type
YFP: yellow fluorescent protein
