# Supplementary figures and images for: Identification of molecular determinants that govern distinct STIM2 activation dynamics
Source: PLoS Biol. 2018 Nov 16;16(11):e2006898. doi: 10.1371/journal.pbio.2006898 (PMC6267984; doi:10.1371/journal.pbio.2006898)

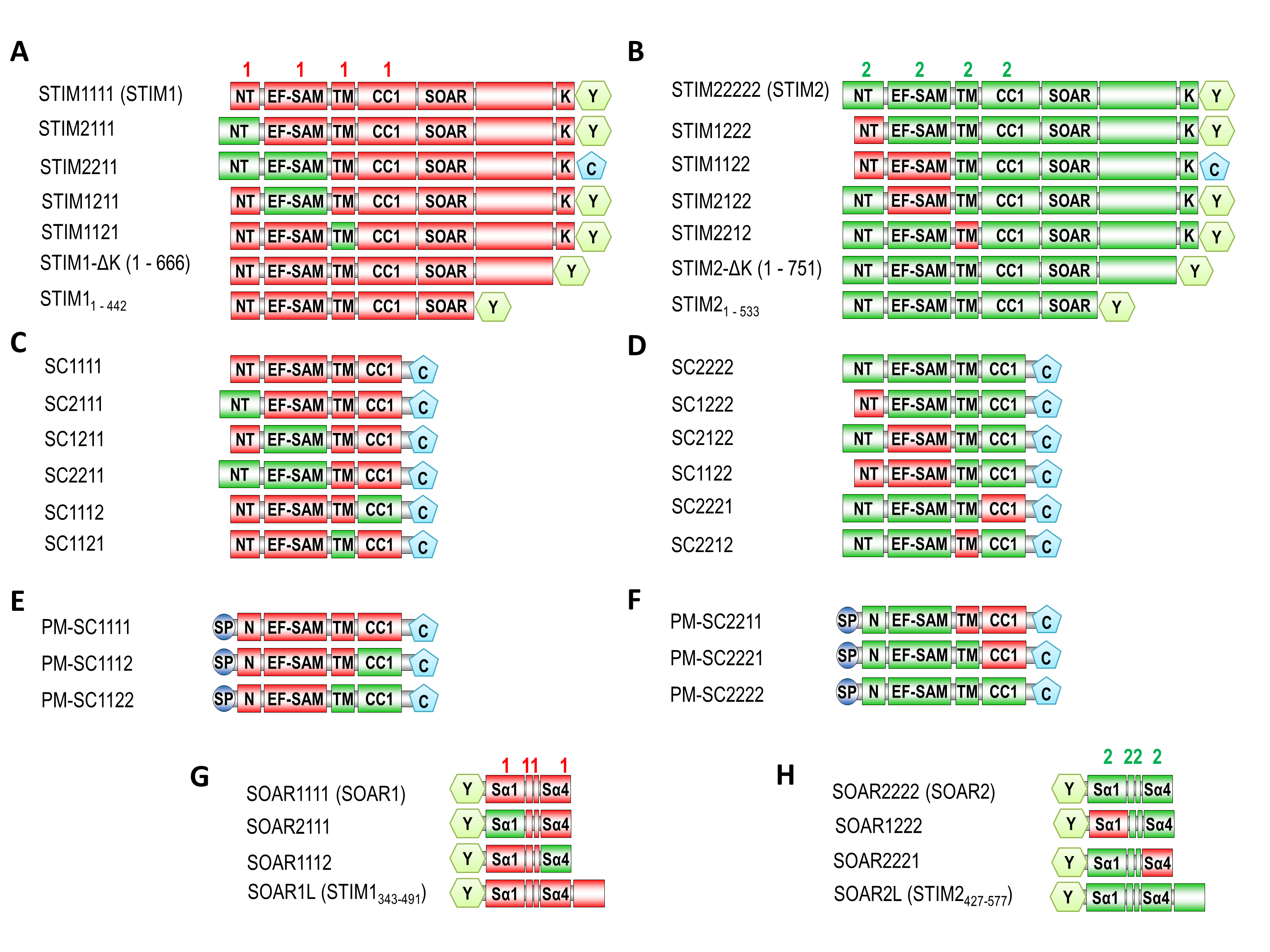

Supplement: S1 Fig — (A–B) Full-length STIM chimeras or constructs with C-terminal deletions. (C–D) SCs and chimeras; (E–F) PM-anchoring SCs and chimeras; (G–H) SOAR constructs and chimeras. C, CFP tag; CC1, coiled-coil 1; CFP, cyan fluorescent protein; EF-SAM, EF-hand and sterile alpha motif domain; K, lysine-rich region; NT, N terminus; PM, plasma membrane; SC, STIM1-CC1 construct; SOAR, STIM-Orai–activating region; SOAR1L, STIM1(343–491); STIM, stromal interaction molecule; sα1–4: α helix 1 to 4 of SOAR region; TM, transmembrane region; Y, YFP tag; YFP, yellow fluorescent protein. (TIF) [file pbio.2006898.s001.tif]

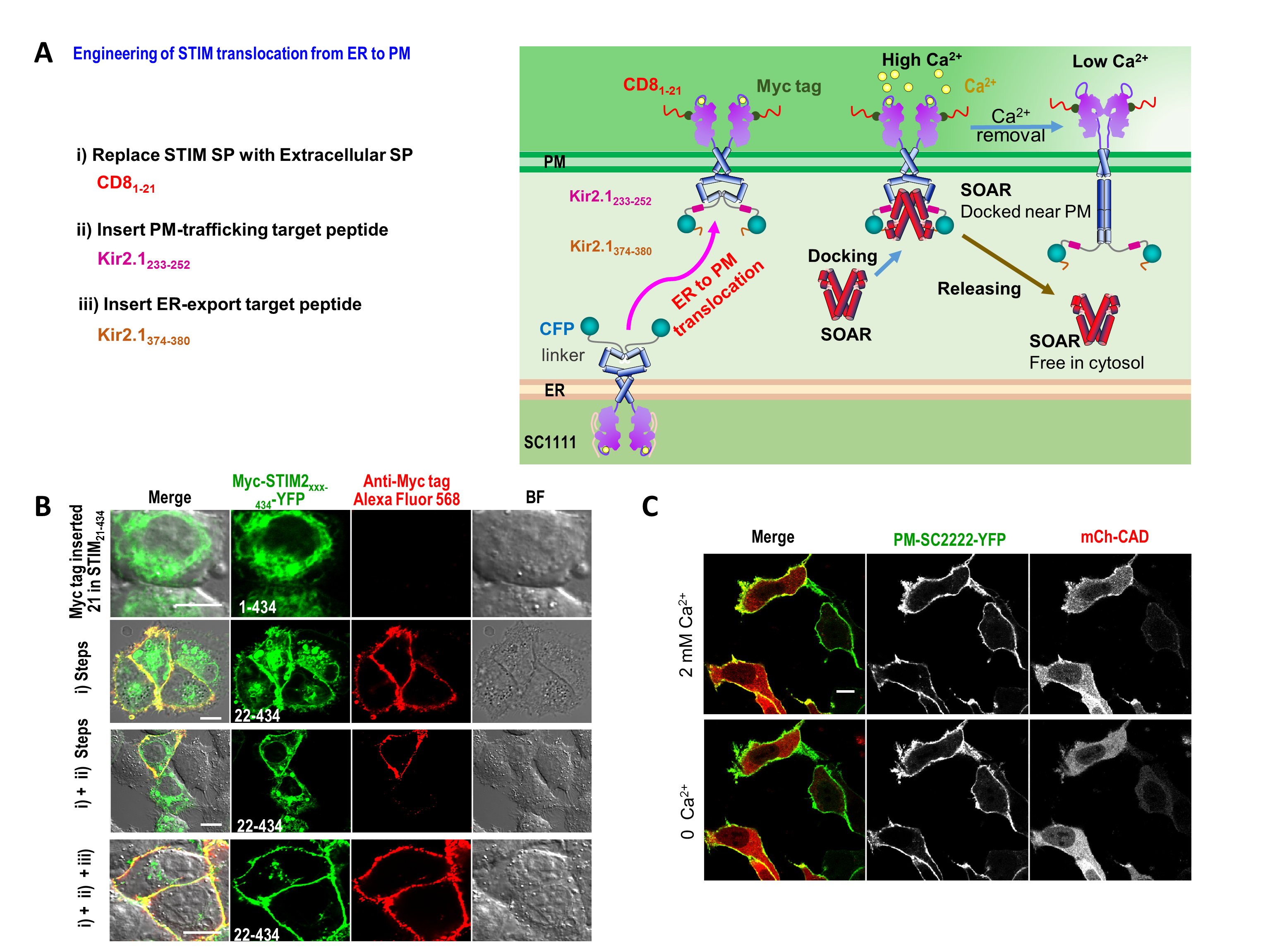

Supplement: S2 Fig — (A) Schematic illustration of the strategies used to force the ER-to-PM trafficking of engineered STIMs. First, a Myc tag was introduced into STIM between SP and EF-SAM to aid the determination of the orientation of the N terminus of STIM. The original ER SP of STIM was replaced by an extracellularly targeting peptide derived from CD8A1-21. To facilitate the ER export of STIM1 and trafficking in the cytosol, PM-trafficking TP (Kir2.1233−252) and ER-exporting TP (Kir2.1374−380) were inserted upstream and downstream of the C-terminal CFP, YFP, or mCh fluorescent tag, respectively. (B) Live-cell immunofluorescence staining of HeLa cells expressing the designed YFP-tagged PM-targeting constructs. Alexa-Fluor-568–conjugated secondary antibody was used to determine the extracellular localization of the Myc tag in nonpermeabilized HeLa cells. (C) Confocal imaging of HeLa cells coexpressing PM-S2222-YFP and mCh-CAD cultured in the 2 mM Ca2+ medium or Ca2+-free medium. Scale bar, 10 μm. CAD, CRAC-activating domain; CFP, cyan fluorescent protein; CRAC, Ca2+-release-activated Ca2+ current; EF-SAM, EF-hand and sterile alpha motif domain; ER, endoplasmic reticulum; mCh, mCherry PM, plasma membrane; SP, signal peptide; STIM, stromal interaction molecule; TP, target peptide; YFP, yellow fluorescent protein. (TIF) [file pbio.2006898.s002.tif]

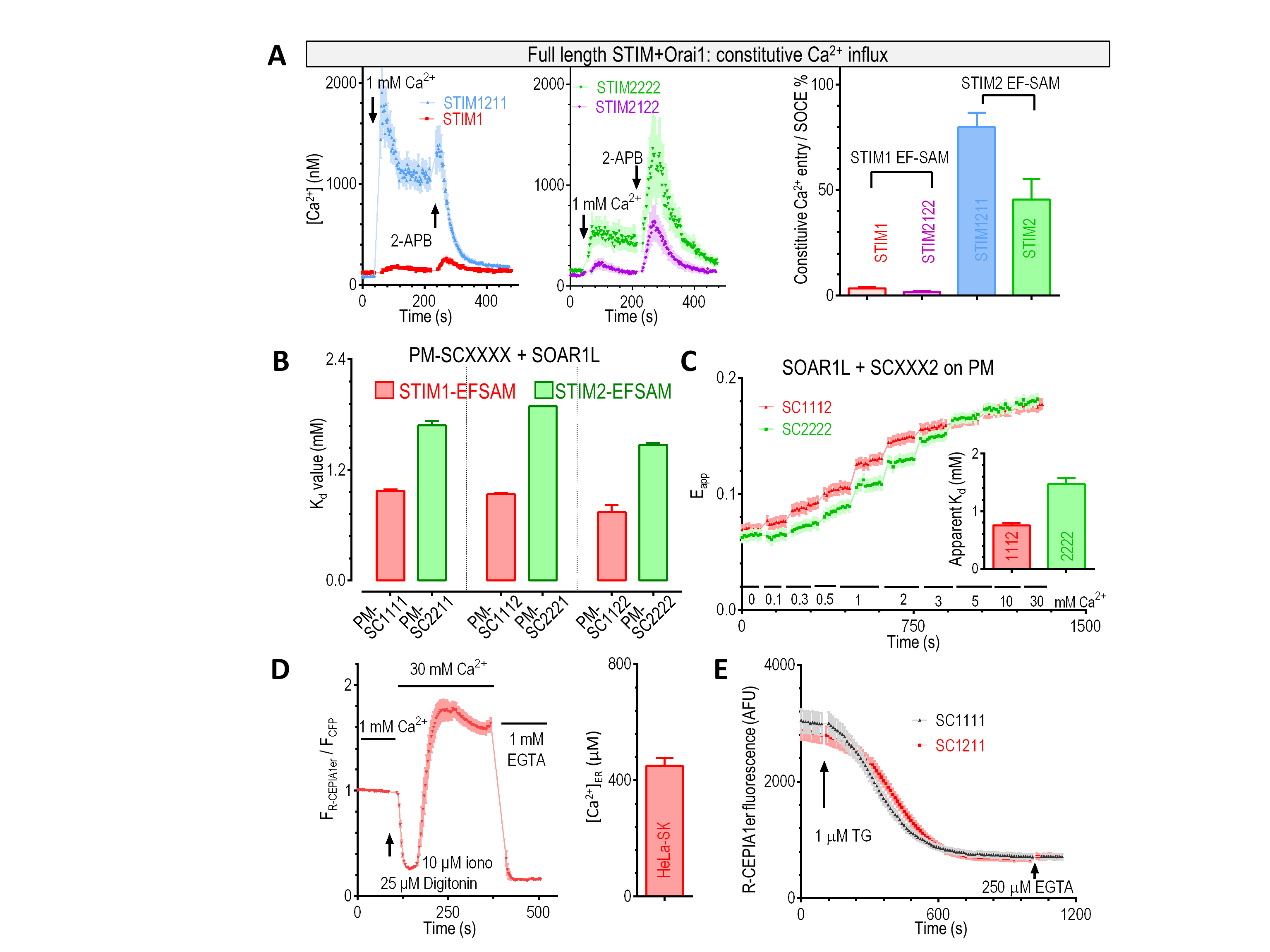

Supplement: S3 Fig — (A) In HEK293-Orai1 stable cells transiently expressing WT STIM or corresponding STIM chimeras with swapped EF-SAM regions, only cells expressing constructs that contain the STIM2 EF-SAM (STIM1211 or STIM2) facilitate a high constitutive Ca2+ influx (blue and green traces); no such constitutive Ca2+ influx was observed in cells expressing constructs harboring the STIM1 EF-SAM (red and purple traces). (B) Statistics showing Ca2+ affinity (mM) of the various PM-anchoring SCs. (C) Some unengineered SCs show some PM-like distribution in approximately 25% of transfected cells. FRET signals between YFP-SOAR1L and PM-localized SC-CFP constructs in response to increases in extracellular Ca2+ concentration in these cells. Left, typical traces; right, statistical analysis of the apparent Kd (n = 5, P = 0.0002). (D) Calibration of the ER Ca2+ levels using R-CEPIA1er and a Ca2+-insensitive ER marker, CFP-Sec61β in HeLa SK cells. Left, a typical trace used for calibration; right, statistics of the ER Ca2+ concentration. (E) In HeLa SK cells coexpressing R-CEPIA1er, YFP-SOAR1L, and SC1111-CFP or SC1211-CFP, ER Ca2+ levels and FRET signals between SCs and SOARL were monitored simultaneously. Typical traces of the rest state and TG-induced responses for R-CEPIA1er signals. Individual numerical values underlying (A)–(E) may be found in S1 Data. CFP, cyan fluorescent protein; EF-SAM, EF-hand and sterile alpha motif domain; ER, endoplasmic reticulum; FRET, Förster resonance energy transfer; HEK293, human embryonic kidney 293 cells; PM, plasma membrane; SC, STIM1-CC1 construct; SK, STIM1 and STIM 2 double knockout; SOAR, STIM-Orai–activating region; STIM, stromal interaction molecule; TG, thapsigargin; WT, wild type; YFP, yellow fluorescent protein. (TIF) [file pbio.2006898.s003.tif]

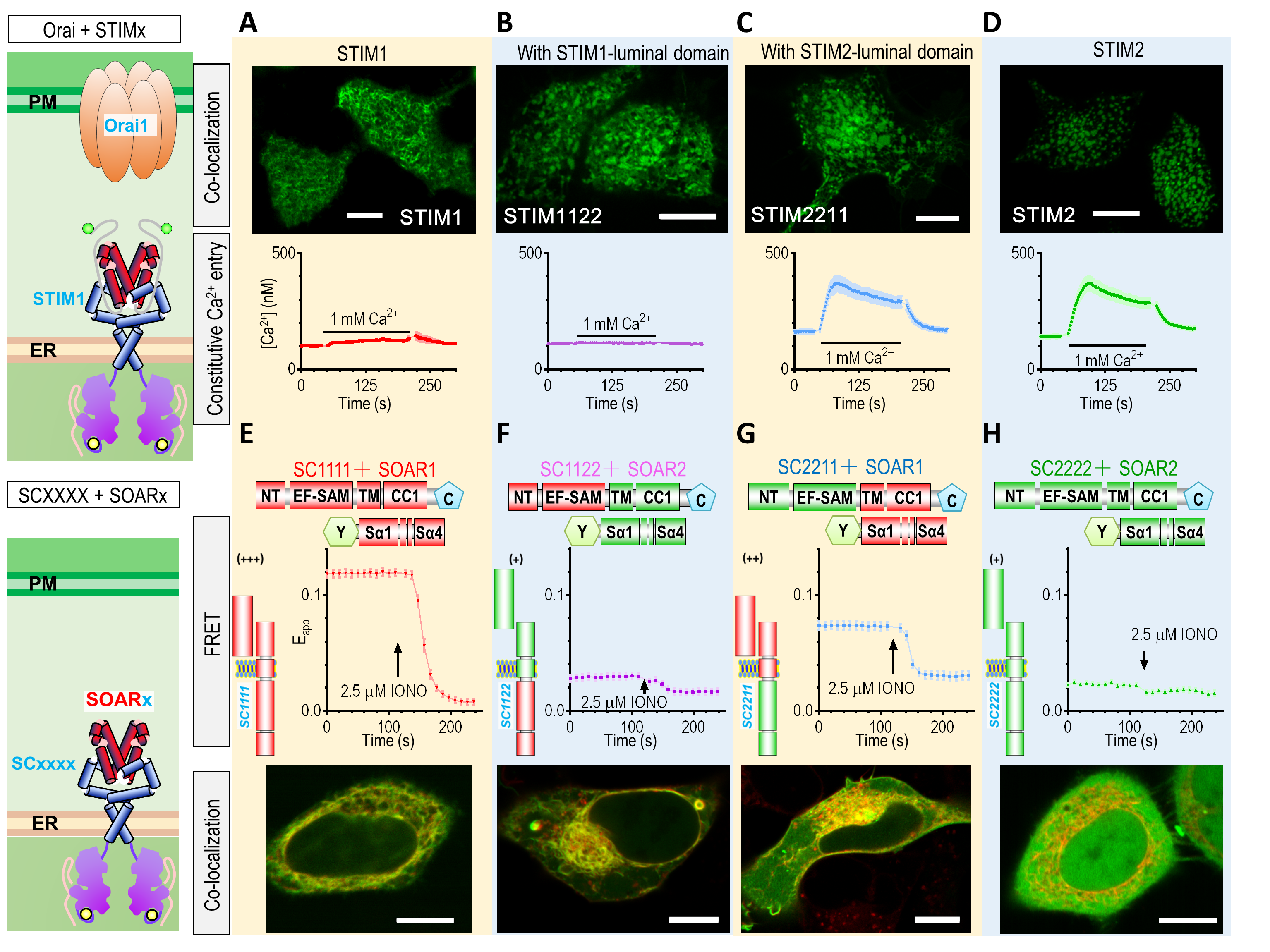

Supplement: S4 Fig — Panels with light yellow background are cells expressing constructs containing the STIM1 cytosolic region; panels with light cyan background are cells expressing molecules containing the STIM2 cytosolic region. (A–D) Comparison of the function of STIM1-YFP (A), STIM2-YFP (B), and the luminal-region–exchanged chimeras, STIM1122-CFP (C) or STIM2211-CFP (D), expressed in HEK293-Orai1-CFP cells or coexpressed with Orai1-YFP in HEK293 WT cells. Left, a diagram of the two coexpressed SOCE components. Top panel: confocal images of the typical cellular distribution of STIM1, STIM2, STIM1122, and STIM2211 at rest (scale bar, 10 μm). Bottom panel: representative traces for a constitutive Ca2+ entry into the Orai1- and STIM-coexpressing cells. (E–G) Comparative analysis of interactions between STIM1-CC1-CFP and YFP-SOAR molecules coexpressed in HEK293 tsA cells, the tsA201 variant of HEK293 cells expressing a temperature sensitive mutant of the SV40 large T antigen. (E) SC1111-CFP+YFP-SOAR1, (F) SC2222-CFP+YFP-SOAR2, (G) SC1122-CFP+YFP-SOAR2, and (H) SC2211-CFP+YFP-SOAR1. The top diagrams show the two coexpressed STIM fragments. Top panel: representative traces of typical FRET signals between WT or chimeric STIM1-CC1-CFP and YFP-SOAR molecules; Bottom panel: confocal images of the typical colocalization of STIM1-CC1-CFP and YFP-SOAR molecules (scale bar, 10 μm). All results are typical of at least three independent repeats, and at least 36 cells were examined for each condition. Individual numerical values underlying (A)–(H) may be found in S1 Data. CC1, coiled-coil 1; CFP, cyan fluorescent protein; FRET, Förster resonance energy transfer; HEK293, human embryonic kidney 293 cells; SC, STIM1-CC1 construct; SOAR, STIM-Orai–activating region; SOCE, store-operated Ca2+ entry; STIM, stromal interaction molecule; tsA, the tsA201 variant of HEK293 cells expressing a temperature sensitive mutant of the SV40 large T antigen; WT, wild type; YFP, yellow fluorescent protein. (TIF) [file pbio.2006898.s004.tif]

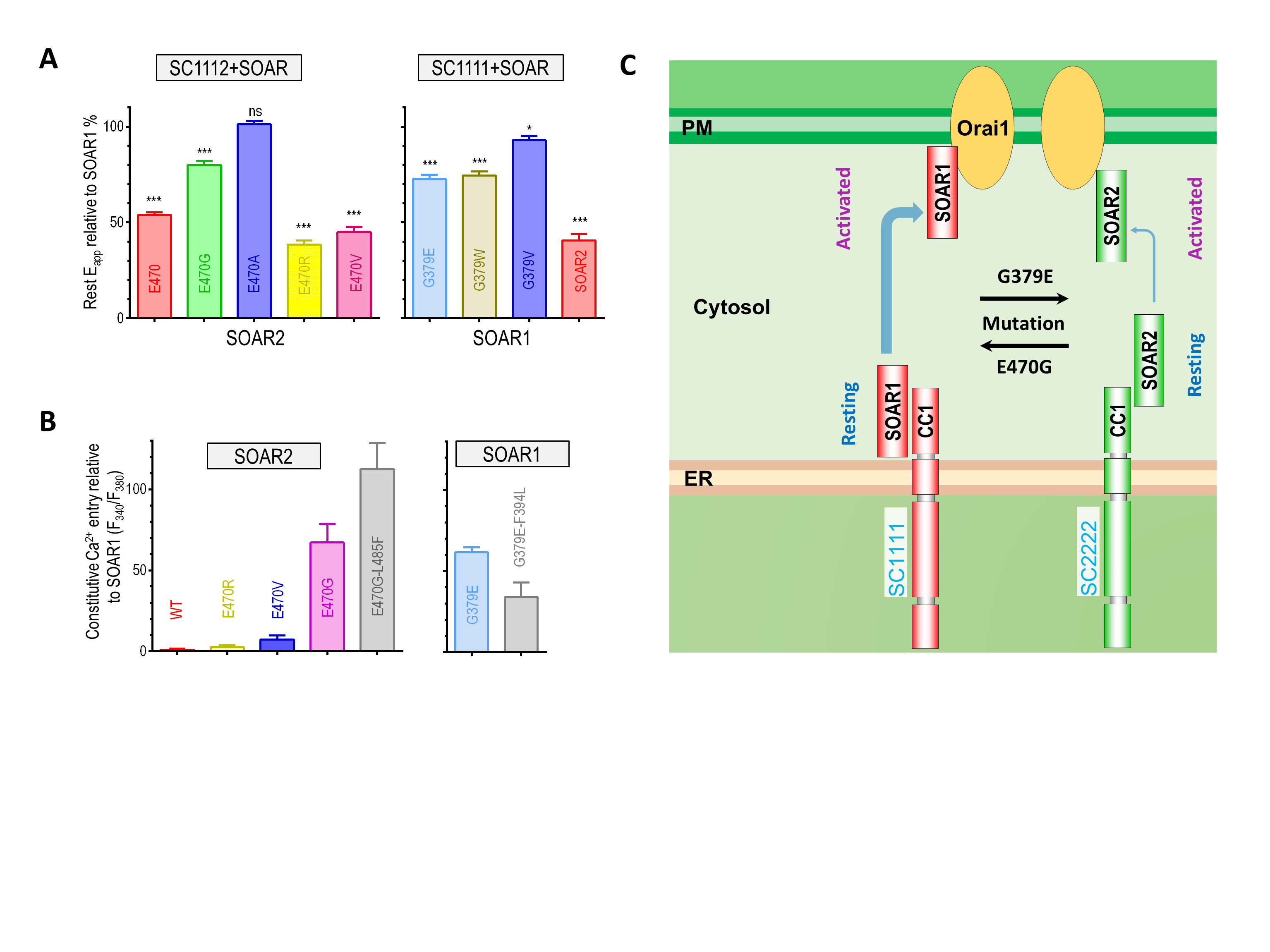

Supplement: S5 Fig — (A) Statistics showing resting FRET signals of SOAR constructs with SC1112 (left) or SC1111 (right). (***P < 0.001; *P < 0.05, t test, n = 3). (B) Statistics of mean constitutive Ca2+ influxes mediated by Orai1 and different SOAR2 (left) or SOAR1 (right) constructs. (***P < 0.001; *P < 0.05, t test, n = 3). Note: all signals were compared with and normalized to those mediated by SOAR1. Individual numerical values underlying (A) and (B) may be found in S1 Data. (C) A simplified, hypothetical cartoon showing how the binding of the SOAR within full-length STIM constructs with its CC1 or Orai1 would define the dynamic range of STIM activation. For simplicity, only CC1 and SOAR domains of the cytosolic region of STIMs were shown. The range of dynamic changes is indicated by the sizes of blue arrows. At rest, CC1 docks SOAR near the ER membrane, preventing it from activating Orai1 on the PM (bottom); after Ca2+ store depletion, SOAR detaches from CC1 and relocates to the vicinity of the PM, where it binds and activates the Orai1 channels. CC1, coiled-coil 1; ER, endoplasmic reticulum; FRET, Förster resonance energy transfer; PM, plasma membrane; SC, STIM1-CC1 construct; SOAR, STIM-Orai–activating region; STIM, stromal interaction molecule. (TIF) [file pbio.2006898.s005.tif]
